# Using targeted enrichment of nuclear genes to increase phylogenetic resolution in the neotropical rain forest genus *Inga* (Leguminosae: Mimosoideae)

**DOI:** 10.3389/fpls.2015.00710

**Published:** 2015-09-17

**Authors:** James A. Nicholls, R. Toby Pennington, Erik J. M. Koenen, Colin E. Hughes, Jack Hearn, Lynsey Bunnefeld, Kyle G. Dexter, Graham N. Stone, Catherine A. Kidner

**Affiliations:** ^1^Ashworth Labs, Institute of Evolutionary Biology, School of Biological Sciences, University of EdinburghEdinburgh, UK; ^2^Royal Botanic Garden EdinburghEdinburgh, UK; ^3^Institute of Systematic Botany, University of ZurichZürich, Switzerland; ^4^School of Geosciences, University of EdinburghEdinburgh, UK; ^5^Institute of Molecular Plant Sciences, School of Biological Sciences, University of EdinburghEdinburgh, UK

**Keywords:** hybrid capture, *Inga*, next-generation sequencing, phylogenomics, population genomics, radiation, targeted enrichment

## Abstract

Evolutionary radiations are prominent and pervasive across many plant lineages in diverse geographical and ecological settings; in neotropical rainforests there is growing evidence suggesting that a significant fraction of species richness is the result of recent radiations. Understanding the evolutionary trajectories and mechanisms underlying these radiations demands much greater phylogenetic resolution than is currently available for these groups. The neotropical tree genus *Inga* (Leguminosae) is a good example, with ~300 extant species and a crown age of 2–10 MY, yet over 6 kb of plastid and nuclear DNA sequence data gives only poor phylogenetic resolution among species. Here we explore the use of larger-scale nuclear gene data obtained though targeted enrichment to increase phylogenetic resolution within *Inga*. Transcriptome data from three *Inga* species were used to select 264 nuclear loci for targeted enrichment and sequencing. Following quality control to remove probable paralogs from these sequence data, the final dataset comprised 259,313 bases from 194 loci for 24 accessions representing 22 *Inga* species and an outgroup (*Zygia*). Bayesian phylogenies reconstructed using either all loci concatenated or a gene-tree/species-tree approach yielded highly resolved phylogenies. We used coalescent approaches to show that the same targeted enrichment data also have significant power to discriminate among alternative within-species population histories within the widespread species *I. umbellifera*. In either application, targeted enrichment simplifies the informatics challenge of identifying orthologous loci associated with *de novo* genome sequencing. We conclude that targeted enrichment provides the large volumes of phylogenetically-informative sequence data required to resolve relationships within recent plant species radiations, both at the species level and for within-species phylogeographic studies.

## Introduction

Evolutionary radiations are ubiquitous and prevalent across disparate plant lineages in diverse geographical and ecological settings (Linder, 2008; Bouchenak-Khelladi et al., 2015; Donoghue and Sanderson, 2015; Hughes and Atchison, 2015; Hughes et al., 2015), yet the trajectories of rapid species diversifications through time and space and the mechanisms and evolutionary processes underlying them remain poorly understood. For example, we know rather little about the evolutionary diversification of species-rich clades in the hyperdiverse neotropical rain forests. Many of these are recent, with extant species dating from the late Miocene (Richardson et al., 2001; Erkens et al., 2007; Särkinen et al., 2007; Koenen et al., 2015), and there are many competing hypotheses, but no consensus, about what has driven rapid species diversification of rain forest clades (Hughes et al., 2013). For instance, the fine-scale hydrological and edaphic mosaic of the Amazon basin has been implicated in the diversification of Protieae (Burseraceae, Fine et al., 2014), dispersal opportunities and rapid evolution of leaf chemistry in response to herbivory have been suggested as important in diversification of the species-rich rain forest legume genus *Inga* (Lavin, 2006; Kursar et al., 2009), and ecological opportunities following the demise of the western Amazonian Pebas wetland system in the late Miocene may have driven recent convergent radiations of Meliaceae (Koenen et al., 2015). In each case within these recent rain forest radiations, current hypotheses of species and population relationships are too poorly resolved to make reliable inferences about trait evolution or trajectories of diversification. Lack of resolution remains the hallmark of current phylogenies for the majority of recent plant radiations.

When lineages have radiated rapidly, establishing a well-resolved and supported phylogeny or population history can be challenging (e.g., Hughes and Eastwood, 2006; Kursar et al., 2009; Fior et al., 2013). Traditional approaches, with datasets of hundreds to several thousands of bases, often fail to resolve recently diverged species radiations, particularly in plants. Much larger DNA sequence datasets are often required to obtain sufficient phylogenetic information to resolve recent events. Large numbers of independent loci are also required to deal with the high levels of incomplete lineage sorting which can occur in rapid radiations (Degnan and Rosenberg, 2009). To produce these larger datasets, many recent studies are using nuclear gene sequences generated via high-throughput next-generation sequencing approaches to provide sufficient resolution (Delseny et al., 2010; Lemmon and Lemmon, 2013; McCormack et al., 2013; Wagner et al., 2013; Grover et al., 2015; Heyduk et al., 2015; Lamichhaney et al., 2015; Stephens et al., 2015a,b).

Throughout the 1990s and early 2000s plant phylogenetics predominantly used plastid data supplemented with a small number of ribosomal nuclear loci (Hollingsworth et al., 2009; Parks et al., 2012). Reliance on plastid data has many limitations. Firstly, because plastid DNA is non-recombining, effort invested in sequencing multiple plastid genes only samples a single shared coalescent history, reducing information content relative to a similar number of unlinked loci. Furthermore, plastid mutation rates are typically low, even in non-coding regions (Wolfe et al., 1987; Small et al., 2004), providing limited resolving power even from kilobases of sequence (e.g., Fior et al., 2013). Using small numbers of loci (either the maternally inherited plastid or nuclear loci) can lead to difficulty in resolving a true species history due to incomplete lineage sorting or hybridization and plastid capture (Chan and Levin, 2005; Stegemann et al., 2012). Sequencing large numbers (hundreds to thousands) of nuclear loci can avoid these problems. As the signal of introgression can persist much longer in organelle data compared to nuclear data (Nicholls et al., 2012), nuclear data are less likely to violate the assumptions made by most phylogenetic algorithms regarding the sources of gene-tree discordance among loci. In addition, the existence of population genomic models that incorporate gene flow between lineages (Frantz et al., 2014; Lohse and Frantz, 2014) make it possible to confirm any hybridization, a relatively common phenomenon in plants (Soltis and Soltis, 2009).

Several methods exist that reduce the complexity of next-generation sequencing approaches by sub-sampling the genomes of study taxa (reduced representation sequencing), providing the benefits of high-coverage data from hundreds or thousands of loci for the many individuals that are typical of phylogenetic datasets, without the cost of whole genome sequencing (Mamanova et al., 2010; Davey et al., 2011; Cronn et al., 2012). These include RAD sequencing, transcriptome sequencing and targeted enrichment (Davey et al., 2011; Good, 2011; Cronn et al., 2012; Egan et al., 2012; Grover et al., 2012; Lemmon and Lemmon, 2013; McCormack et al., 2013; Grover et al., 2015; Heyduk et al., 2015; Stephens et al., 2015a,b). Criteria for deciding which approach to use commonly include maximizing useable data for as many individuals as possible whilst minimizing cost. We focus here on targeted enrichment, a technique that uses a hybridization reaction involving custom-designed short RNA or DNA probes (“baits”) in solution or on an array to capture thousands of target loci with sequences similar to the set of baits from fragmented genomic DNA libraries (Mamanova et al., 2010; McCormack et al., 2013).

Targeted enrichment was originally developed as a way of screening specific regions of the human exome but its advantages of moderate cost, specificity, low input amounts of genomic DNA and ability to target large numbers of markers make it highly applicable to phylogenomic and population genomic studies of non-model organisms. This technique also has the advantage that multiple reads from the target loci can be assembled into longer sequences, thus providing alignments more likely to contain multiple variable sites that are amenable to gene-tree based phylogenomic or population genomic analyses (McCormack et al., 2013; Lohse and Frantz, 2014). It has wide potential use, from intra-specific population studies, typically of SNP variation (e.g., Salmon et al., 2012; Winfield et al., 2012; Zhou and Holliday, 2012; Tennessen et al., 2013) to deeper-level phylogenomics (e.g., Parks et al., 2012; Stull et al., 2013; Mandel et al., 2014; Weitemier et al., 2014; Grover et al., 2015; Heyduk et al., 2015; Stephens et al., 2015a,b).

Enrichment through hybridization will often capture paralogs and even divergent loci that share a conserved domain. Paralogs can be dealt with though careful selection of bait sequences, through conservative assembly of sequence and/or through phylogenetic analysis. A draft genome can be used for bait design, minimizing paralog capture by avoiding multi-copy loci or those in gene families (for example Weitemier et al., 2014; Grover et al., 2015). However, generating a sufficiently informative genome can incur substantial time and cost, particularly in plants with large and complex genomes. Even with prior genomic information, capturing paralogs may be unavoidable in plants because of widespread whole genome duplication events, for example those preceding the origin of angiosperms and in many major angiosperm clades (Vanneste et al., 2014; Cannon et al., 2015). An alternative approach for bait design in non-model groups is to use transcriptomes of the study species or related species (Heyduk et al., 2015; Stephens et al., 2015a,b) We used *de-novo* assembled transcriptome data from three species across our radiating lineage to select genes and design target baits. This approach has the advantage of relatively low cost and uses sequence variation, expression level variation and annotation from related model species to guide the choice of loci. Using reciprocal best hit blastn algorithms on data from multiple transcriptomes helps to reduce the number of selected loci belonging to multi-copy gene families. However, selecting low-copy nuclear genes from transcriptomes is not as straightforward as it is from full genomes. Transcriptomes only contain gene sequences expressed in the tissues sequenced, so in many cases where a single orthologous copy is identified through a reciprocal best hit process, paralogous non-expressed copies or non-functional pseudogene copies may still be present within the genome. The absence of positional information and intron sequences in expression data can also make it difficult to distinguish recently diverged paralogs from allelic variation within a single locus. This leads to an *a priori* expectation that paralogous loci may be sequenced from enriched genomic libraries. Therefore, we have also relied on conservative mapping of captured sequences and phylogenetic analysis to eliminate paralogy problems. Our pipeline exploits the high coverage per bait produced by targeted enrichment to output a very conservatively-called consensus for the paralog with the highest and best quality coverage. We then use outgroup information to test that orthologous sequences are obtained across accessions and filter out loci with apparent paralogs.

Here we apply this targeted enrichment method to generate data for phylogenomic and population genomic analyses of the species-rich, ecologically important and abundant neotropical tree genus, *Inga* (Leguminosae: Mimosoideae, Pennington, 1997). *Inga* is widely distributed from Argentina to Mexico, is one of the most diverse neotropical tree genera with ~300 species, and has consistently high local abundance and species diversity in rain forests across its range. It therefore provides an excellent system in which to investigate the origin and maintenance of rain forest hyperdiversity (Richardson et al., 2001; Kursar et al., 2009; Dexter et al., 2012). Current phylogenetic hypotheses for *Inga* (based on the plastid DNA markers *trnL-F, trnD-T, psbA-trnH, rps16, rpoC1*, and *ndhF-rpl32* and the nuclear ribosomal 5.8S subunit and internal transcribed spacers, *ITS1* and *ITS2*) fail to resolve many relationships (Richardson et al., 2001; Lavin, 2006; Kursar et al., 2009; Dexter et al., 2010), especially amongst closely related species, hindering understanding of the factors driving diversification. This is thought to be due to the recent, rapid radiation of *Inga*—all the extant species are estimated to have arisen during the past 2–10 million years (Richardson et al., 2001; Lavin, 2006) so are separated on a phylogeny by relatively short branches, and many species have very large effective population sizes (ter Steege et al., 2013). This increases the chances of incomplete lineage sorting, whereby gene copies fail to coalesce before deeper speciation events (Pamilo and Nei, 1988). Population-level analysis is critical to understanding the patterns of evolution in this group. While current data suggest that hybridization has not played an important role in this lack of resolution (see Koptur, 1984), there are few data available about the possible extent of hybridization and past or current gene flow between species and between populations.

We use transcriptomes from three *Inga* species to design a bait set for 264 loci, and develop strategies for the removal of potential paralog sequences. We examine the utility of the resulting datasets in (i) resolving phylogenetic relationships among 22 *Inga* species, and compare the resulting phylogenetic hypothesis with those obtained from plastid and nuclear loci routinely used for phylogenetics; and (ii) resolving relationships among four geographically diverse populations of a single *Inga* species, and provide power analyses that explore the numbers of loci required to discriminate between alternative population histories over two contrasting timescales.

## Materials and methods

### Transcriptome generation

We generated transcriptomes from three *Inga* species to use as the basis for selecting genes and designing baits for targeted enrichment (Supplementary Table 1). To explore sequence divergence at various phylogenetic levels in *Inga*, we selected two species that existing phylogenetic data (Dexter et al., 2010) suggest are closely related (*I. sapindoides, I. spectabilis*) and another more distantly related species (*I*. *umbellifera*). Expanding leaves from one tree of each species were harvested on Barro Colorado Island, Panama, immediately cut into small strips approximately 2 mm wide by 10 mm long and preserved in RNAlater. Total RNA was extracted using Qiagen RNeasy kits with buffer RLC (*I. sapindoides*) or Invitrogen Plant RNA Reagent with 8 M lithium chloride precipitation (*I. spectabilis* and *I. umbellifera*), and DNase treated with Turbo RNase-free DNase (Invitrogen). Libraries were made from the total RNA using Illumina's TruSeq RNA Sample Prep kits and ~50 M reads (100 bp paired end sequence) generated per library on an Illumina HiSeq machine.

Reads were quality trimmed used Trimmomatic v0.30 (Bolger et al., 2014) with settings: ILLUMINACLIP:TruSeq3-PE.fa:2:30:10 LEADING:3 TRAILING:3 SLIDINGWINDOW:4:15 MINLEN:36. Trinity (release r20120608, Grabherr et al., 2011) was used for *de-novo* assembly of each of the three libraries individually using the default parameters. Reads from all three libraries were also Trinity assembled together into a single *Inga* reference transcriptome, also using default parameters. Bowtie2 v2.0.2 (Langmead and Salzberg, 2012) was used to align the reads from each species back to this reference *Inga* transcriptome to give comparative expression levels in reads per KB of contig per million reads mapped (RPKM). Annotations (Gene Ontology (GO) terms, Enzyme Commission (EC) numbers and gene structures) for this *Inga* reference transcriptome were derived through comparison using blastx searches to the proteomes of the model species *Glycine max, Lotus japonicus* and *Arabidopsis thaliana*. To assess completeness of transcriptomes and potential paralogy within them, the Core Eukaryotic Genes Mapping Approach (CEGMA v2.5, Parra et al., 2007) was run on assemblies of each individual species and the combined reference transcriptome. The longest element of each Trinity component group was selected for this to avoid inflating results by including multiple isoforms of a gene.

### Target loci selection

We performed reciprocal best blast hits using blastn and a cut-off of 1e-80 to identify 12311 reference contigs with clear orthologs in each species. This is a conservative search and will tend to lose sequences from multigene families that may not blast back to the original gene family member. For the genes passing this reciprocal best blast hit criterion we created a database using the annotation described above for the reference transcriptome along with RPKM for each species mapped to the reference transcriptome ortholog, descriptions of the *Glycine* and *Arabidopsis* orthologs including GO terms and EC numbers, intron-exon structure of the *Glycine* and *Lotus* orthologs, and percentage identity between each pair of *Inga* species (Supplementary Table 1). We queried this database using a Python script to produce the following three sets of sequences, the latter two of which reflect wider research goals relating to the genetics of secondary chemistry in *Inga*, although we also examine their phylogenetic utility here.

- *Phylogenetically useful genes* (54 loci). These genes showed a useful level of potentially phylogenetically informative substitutions amongst species, with a percentage nucleotide identity between the closely-related species pair *I. spectabilis* and *I. sapindoides* of less than 98.5%. They were also selected to have only a single sequence per *Glycine* ortholog (hence selecting against known multi-copy gene families, although the possibility that these loci are multi-gene in *Inga* clearly cannot be ruled out), contain between one and three introns and less than 1 kb of total intron sequence in the corresponding region of the *Glycine* ortholog (thus potentially facilitating ease of assembly post-sequencing), and have no more than 45% or no fewer than 25% of the reads for the reference sequence coming from any one of the three sampled species.
- *Differentially expressed genes* (109 loci). To identify enzymes differentially expressed between species we summed the reads in each transcriptome for contigs annotated with any one EC number and created a list of EC numbers with more than 75% of their reads from a single species. Reference transcriptome sequences annotated with these EC numbers were identified and filtered by their presence in the reciprocal best blast hit database or, for those not expressed in all species, a blast match to *Glycine* of less than 1e-40, and between one and three introns in the matching region of the *Glycine* ortholog.
- *Secondary synthesis genes* (113 loci). Forty-three enzymes potentially important in the production of secondary defensive chemicals in *Inga* were identified by Tom Kursar (University of Utah). We used the Brenda database (Schomburg et al., 2013) to retrieve sequences for each of these, which were then blasted at Phytozome (Goodstein et al., 2012) to gather sets of legume sequences for each enzyme. These were blasted against the reference transcriptome to identify 113 *Inga* loci.

Putative exons in the full set of 276 target loci were identified using blastx searches against a reference database of proteins from *Medicago, Glycine*, and *Arabidopsis*, with each exon treated as a separate target sequence so that individual baits were not located across intron/exon boundaries. In total 907 putative exons were identified. These sequences were used to design a MYbaits bait library (MYcroarray, Ann Arbor) to give 3 × tiling of 120 bp RNA baits for each target exon.

### Library generation and hybridization

Next-generation sequencing data were obtained for a set of 23 *Inga* and one *Zygia* accessions (hereafter called the comparison set) that had previously been sequenced for plastid/ITS loci. This sampling incorporated 22 *Inga* species spanning the phylogenetic diversity of the genus (e.g., Kursar et al., 2009) and covering the morphological diversity and currently recognized taxonomic sections of *Inga*, plus one species of *Zygia* as an outgroup (Dexter et al., 2010, Supplementary Table 2). In order to assess the utility of these data to resolve relationships within species, 19 individuals from a widespread species that occurs across Amazonia, *I. umbellifera*, and two individuals from its close relative, *I. brevipes*, were also sequenced (see Supplementary Table 2). *Inga umbellifera* individuals came from four spatially separated populations, one of which contains two chemically-distinct sub-populations. A technical replicate was also included to assess the amount of sequencing and assembly error in our analysis, which involved entirely separate library preparation, enrichment, and sequencing steps for two aliquots of DNA from one *I. umbellifera* individual.

DNA extractions from a 15 mm by 8 mm section of silica-dried leaf material were performed using the Qiagen DNeasy Plant Mini kit with several modifications. These modifications were (i) three tissue disruptions for 40 s at 20 Hz; (ii) a 30 min incubation at 65°C in buffer AP1 and RNaseA followed by addition of 1 μL of RiboShredder RNase blend (Cambio) and a further 20 min incubation at 37°C; and (iii) a revised elution process designed to remove degraded DNA fragments and maximize recovery amounts in a smaller 65–68 μL volume (addition of 50 μL of buffer EB to the membrane, incubate for 20 s, spin for 1 min at 8000 rpm and discard flow through, then add 43 μL buffer EB to membrane, incubate for 5 min at room temperature, spin for 1 min at 8000 rpm, re-apply this flow-through to membrane for a second 5 min incubation, spin for 1 min at 8000 rpm, apply 25 μL of fresh buffer EB, incubate for 5 min at room temperature, spin for 1 min at 8000 rpm).

Single-indexed libraries were made for each sample using Illumina's TruSeq Nano DNA LT Sample Preparation kit, following the 350 bp insert size protocol with the only alteration being that shearing was performed on a Diagenode Bioruptor Plus (low power, 8 cycles of 30 s on/90 s off) rather than a Covaris sonicator. Final libraries were diluted to 10 nM and equimolar amounts added into five pools, two with 12 accessions each and three containing 8 accessions each. Hybrid enrichment was performed on these five pools using a single capture reaction per pool following the MYbaits v2.3.1 protocol, with between 17 and 21 h of hybridization, a high stringency post-hybridization wash and a final amplification involving 13 PCR cycles using Herculase II Fusion polymerase. Post-capture library pools were quantified using Qubit (Life Technologies), their size distributions assessed on a Bioanalyser (Agilent Technologies), then pools were diluted to 10 nM. Equimolar amounts of the two 12-accession pools were combined into one master pool and the three 8-accessions pools combined into a second master pool; these two pools were sequenced by the Edinburgh Genomics facility in two runs of an Illumina MiSeq using 250 bp paired-end reads.

### Assembly of captured sequence and removal of paralogs

Read quality was checked using FastQC (http://www.bioinformatics.babraham.ac.uk/projects/fastqc/). Raw reads were trimmed using Trimmomatic v0.30 and Cutadapt v1.4.1 (Martin, 2011) and unpaired reads removed from the resultant files using custom shell scripts. In order to simplify the assembly process for each library, Bowtie2 v2.0.2 was used to align the reads back to the 276 target loci contigs from the reference transcriptome. The consensus sequence for each locus was extracted using bcftools from the SAMtools v0.1.18 package (Li et al., 2009). Consistent with the presence of paralogous loci observed in the CEGMA analysis (see Section Results below), visual inspection of the bam files indicated that reads from obviously different genomic loci mapped back to each target locus when using the default alignment parameters in Bowtie2 in local mode. This introduced spurious base calls in consensus sequences (and potentially chimeric consensuses) for each accession, and depending on the relative mapping efficiencies of different paralogs, consensus sequences could be dominated by one paralog in some accessions and a different paralog in other accessions. This introduced noise into subsequent phylogenetic analyses, potentially contributing to unusually long terminal branches (see below and Supplementary Figure 1A). We undertook three steps to optimize the mapping procedure and consensus calling in order to minimize inclusion of paralogous sequences.

Firstly, a small number of known duplicate loci (or members of conserved gene families) were present in the target set of 276 loci, so these were removed, resulting in a final set of 264 reference loci. Secondly, the threshold for the alignment score used by Bowtie2 to determine whether a read aligned to the reference was increased. This threshold is calculated as [constant + 8^*^ln(x)] where x = read length. The default value for the constant is 20, so we assessed the effect of increasing this in units of 40, from 20 to 420. The impact of this increase was measured using the change in the percentage of sites called as different from the reference and the change in the standardized quality of those variant sites (expressed as the average quality of variant sites divided by the average quality of non-variant sites, under the expectation that the average quality of non-variant sites gives a measure of the overall decrease in the quality of any base call in response to a decrease in the number of reads mapping to any particular site). This procedure was repeated for three accessions. The change in percentage of variant sites decreased at a steady rate until alignment constant values of about 300, after which it began to decrease rapidly, reflecting a decrease in variant site calls due to a gradual decrease in the number of reads from paralogous loci mapping until the point at which sufficiently few reads mapped that the ability to call variants with confidence was impacted (Supplementary Figure 2). The change in standardized quality of variant calls increased up to scores of about 260, at which point it leveled out, again potentially reflecting the improvement associated with fewer paralogs mapping to any particular target locus (Supplementary Figure 2). Given these consistent patterns across the three test accessions, mappings were re-done for the full set of accessions using a conservative mapping threshold value of 320.

The third optimization step involved removing low quality base calls from the resultant vcf file. The effect of increasing the minimum quality score required for retention in the vcf file from 24 to 48, in increments of 3, was assessed for both the total number of bases and the number of variant calls excluded. There was a linear response to increasing this threshold in the number of variants excluded, but an increase in the rate at which the total number of bases excluded changed with minimum score for scores greater than approximately 33 (Supplementary Figure 3). As above, we took a conservative approach and excluded all base calls with a quality less than 36. The small number of indel calls present were also excluded, as these were also typically of low quality.

Having exported a multi-fasta file containing the consensus target sequences for each accession, ambiguity codes were converted to Ns. Whilst this may have removed true heterozygous calls, it also removed spurious heterozygote calls due to any co-mapping of paralogs to the reference that may have passed through previous filters. Multi-fastas for each accession (containing sequences for each target locus) were converted into separate multi-fasta files for each locus (each containing sequences for every accession). If data were not present for any particular locus by accession combination then the output written to the new multi-fasta file for that accession was a string of 260 Ns. This conversion process was done for two sets of accessions—the comparison set of 24 accessions with Sanger data to allow direct comparison of the two datasets, plus the larger set of all 46 accessions including the population-level sampling within *I. umbellifera* and the technical replicate. These multi-fastas were aligned using MAFFT v7.130b (Katoh et al., 2009). Poorly aligned or gap-rich regions were removed with trimAl v1.2 (Capella-Gutiérrez et al., 2009) using the -strict setting, and the remaining fasta alignments were converted to nexus files. A plastid alignment was generated using the same method but with a reference of plastid sequence from *I. leiocalycina* (Dugas et al., in review) Custom scripts used for mapping optimization and the subsequent manipulation of consensus sequences are available at https://github.com/ckidner/Targeted_enrichment.git.

Despite using the more stringent mapping settings above, an initial assessment of individual-locus neighbor-joining gene trees indicated that for some loci multiple paralogs were still present in the final nexus alignments. Two further screening steps were undertaken to remove these loci, based on coverage (excluding loci where reads originating from different members of gene families may have mapped to a single highly-conserved domain within the reference) and levels of variation (excluding loci where reads mapped to one paralog in some accessions and another paralog in the remaining accessions). Firstly, for each accession the coverage was calculated both for the sequence obtained for each locus and as an average across loci. An alignment was then excluded from further analyses if, for more than a third of accessions in that alignment, the coverage for any accession was greater than three times the average coverage for the same accession (implying data from multiple loci being mapped). The second screening of loci took advantage of the fact the dataset contained an accession from the outgroup *Zygia*, which is genetically very divergent relative to the variation observed within *Inga* (Kursar et al., 2009; Dexter et al., 2010). PAUP^*^ v4.0b10 (Swofford, 1998) was used to calculate the numbers of parsimony informative and variable sites within the alignment for each locus, first when including *Zygia* and then when considering only the ingroup *Inga* accessions. Assuming the duplication event leading to the extensive paralogy of genes observed within *Inga* occurred before the common ancestor of *Inga* and *Zygia*, if one paralog is sequenced and mapped in some accessions (including *Zygia*) and another mapped in the remaining accessions, then little or no change in the proportion of variable sites should be observed for alignments with or without *Zygia* since the major source of variation among sequences reflects the ancestral duplication event and the paralog sampled from *Zygia* would be nested within one side of the resulting phylogeny. In contrast, if only a single ortholog is mapped in all accessions, removing the outgroup *Zygia* should have a large impact on the number of variable sites. Following this logic, we examined the distribution of the change in variation on exclusion of *Zygia* (Supplementary Figure 4) and, in combination with visual assessment of individual gene trees, determined that loci should be excluded when the percentage change in the proportion of variable sites was 8% or less than the total variation in the alignment including *Zygia*.

In order to assess the impact these changes in mapping stringency and screening of loci had on branch lengths, in particular the length of terminal branches, neighbor-joining analyses were conducted in PAUP^*^ using the comparison set of 24 accessions and the four sets of loci passing the different screening stages: default mappings (275 loci); stringent mappings including all loci (264 loci); stringent mappings excluding high coverage loci (248 loci); and stringent mappings excluding both high coverage loci and those loci where removing *Zygia* had minimal impact on variation (194 loci). Each set of loci were concatenated and a neighbor-joining tree constructed based upon a distance matrix incorporating substitution model parameter values estimated from the best-fit model (TVM+I+G) selected by AIC in jModeltest v2.1.7 (Darriba et al., 2012). Branch lengths were then assessed under this substitution model using the “describe trees” option in PAUP^*^.

### Species-level phylogenetic reconstruction from targeted enrichment data

Two approaches were used to analyse the data for the comparison set of 24 accessions—concatenation of all the data and a gene tree/species tree analysis. For the concatenation approach, alignments for the final screened set of 194 loci were combined, and sites where data were missing for more than half of the 24 taxa were removed using trimAl v1.2 (using the option -gt 0.5). The sequence data were analyzed without assuming that they evolved in a clock-like fashion in a Bayesian framework implemented within the program MrBayes v3.2.2 (Ronquist et al., 2012), considering the whole concatenation as a single partition and applying a GTR+I+G substitution model. Two independent runs (each with 4 chains) of 20 million generations were used, sampling every 2500 generations, and the tree topology was estimated using the combined sample from the last 4 million generations of both runs. Effective sample sizes of parameters within this sample were all >2000.

The appropriateness of a molecular clock was tested in MrBayes by running two further analyses on the concatenated data, one assuming a strict clock and the other assuming a relaxed clock (estimated using the IGR model). A strict clock was very strongly rejected (twice the difference in the natural logarithm of the harmonic mean of model likelihoods (2ΔlnHML = 809.4, see Kass and Raftery, 1995 for cutoff values), whereas there was essentially no difference between a relaxed clock model and a no-clock model (2ΔlnHML = 2.6 in favor of a no-clock model, although likelihood distributions for both models were almost completely overlapping). Given this, the impact upon tree topology and support of running a model explicitly incorporating a molecular clock (hence producing ultrametric trees) was assessed using BEAST v2.1.3 (Bouckaert et al., 2014) under a GTR+I+G substitution model, relaxed log-normal clock and Yule speciation prior. Three independent runs were used, each of 20 million generations and sampled every 2500 generations, with tree topology estimated using the combined sample from the last 4 million generations of each run. Effective sample sizes of estimated parameters within this sample were all >100.

Since concatenation of multilocus data imposes a single tree topology and hence disregards coalescent variation among loci (Maddison, 1997; Edwards, 2009), two species tree approaches were also used to explore effects of between-locus variation in coalescent history. The first was implemented using the program ASTRAL (Mirarab et al., 2014) using a set of gene trees as the input for species tree estimation (rather than co-estimating both gene and species trees, Leaché and Rannala, 2011). To create bootstrapped input trees for ASTRAL, the software RAxML (Stamatakis, 2014) was run on alignments of the 194 loci in the final dataset, using a GTR + GAMMA model and the rapid bootstrapping option with 100 bootstrap replicates for each locus. ASTRAL was run under the multi-locus bootstrapping option (Seo, 2008) with 100 replicates.

An alternative approach used the ^*^BEAST algorithm (Heled and Drummond, 2010), for species-tree analysis within BEAST v2.1.3. To save computing time, rather than the full set of loci we used the 60 most variable remaining after screening for paralogs (proportion of variable sites ranging from 6.1 to 12.2%) that also had data for every accession. Alignments for these loci were trimmed to remove sites where data were missing for more than half the accessions. The type of substitution model for each locus was selected through AIC in MrModeltest v2.2 (Nylander, 2004, see Supplementary Table 3), with parameter values for these models, relaxed clock rates and gene tree topologies estimated independently for each locus, as well as a species tree topology. All target loci are from the nuclear genome so had ploidy set to 2, and a Yule speciation model was used for the species tree prior. Both linearly changing and constant population size models were tested, with a linearly changing model giving a marginally better fit. Three independent runs were performed, each of 200 million generations sampled every 25,000 generations, with the sample for species tree estimation taken from the last 40 million generations of each run, within which nearly all parameters had an effective sample size of >100.

### Species-level phylogenetic reconstruction from current plastid and ITS data

Previously published work on *Inga* phylogenetics (Richardson et al., 2001; Kursar et al., 2009; Dexter et al., 2010) used data from up to seven non-coding plastid loci (ndhF-rpl32, psbA, rpoC1, rps16, trnL-F, rpl32-trnL, and trnDT) and the 5.8S subunit and flanking internal transcribed spacers ITS1 and ITS2 of nuclear ribosomal DNA. To allow direct comparison with our targeted enrichment approach, we used Sanger-derived sequence data from these eight loci to construct a phylogeny for the comparison set of taxa used for targeted enrichment. Where possible, Sanger-sequenced data were included from the same accession used for next-generation sequencing; if data were missing for particular loci then Sanger-sequenced data were used from another accession of the same species. These sequence data were generated through PCR amplification following protocols in Richardson et al. (2001) and Kursar et al. (2009), with amplicons sequenced using BigDye terminator chemistry. Base calling was checked by eye and alignments created using MUSCLE v3.8.31 (Edgar, 2004).

As with the targeted enrichment data, both concatenation and species tree approaches were used to analyse the Sanger sequence data. Firstly, all the data were analyzed as a single concatenation within MrBayes v3.2.2, with partitioning by locus to allow for independent substitution models (using the best model selected through AIC in MrModeltest v2.2; see Supplementary Table 3) but assuming no molecular clocks. Two independent MCMC chains were run for 6 million generations, each sampled every 1250 generations with the final 1.2 million generations of each used for estimating tree topology. Runs of this length sampled at this frequency produced effective sample sizes >100 for parameters. A second concatenated analysis (with the data partitioned by locus) was run in BEAST v2.1.3, using the best substitution model for each locus, but also assuming independent relaxed lognormal clocks for each locus and a Yule speciation model tree prior. Two independent MCMC chains were run for 20 million generations, each sampled every 2500 generations with the final 4 million generations of each used for estimating tree topology. Runs of this length sampled at this frequency produced effective sample sizes >200 for parameters of interest.

Secondly, a species tree analysis using the ^*^BEAST algorithm within BEAST v2.1.3 was performed. Since the plastid genome represents a single non-recombining unit, the Sanger data were divided into two partitions (nuclear ITS and a concatenation of all plastid loci), with independent relaxed clocks, substitution models and gene trees estimated for both partitions as well as an overall species tree. The appropriate type of substitution model was estimated in MrModeltest v2.2, the ploidy for the plastid and nuclear partitions was set to 0.5 and 2 respectively, and a Yule speciation model used for the species tree prior. Two different population models (linear change and constant population size) were tested and as with the targeted enrichment data a linearly changing model gave a marginally better fit. Two independent chains were run in the final analysis for 50 million generations, sampled every 6250 generations, with samples taken from the last 10 million generations used for tree estimation. Effective sample sizes of all parameters within this sample were >100.

### Population-level inference from targeted enrichment data

The utility of the targeted enrichment data for resolving populations and evaluating species limits was assessed in a phylogenetic context using a concatenated analysis involving all 46 accessions (the 24 comparison set accessions, plus the population-level sampling within *I. umbellifera*, its close relative *I. brevipes* and the technical replicate). After applying the same coverage and variation rules (with the same empirically-derived cutoff values) for screening loci as outlined above, 168 loci were concatenated and sites with missing data for more than half the accessions were removed using trimAl v1.2. The concatenated dataset was run in MrBayes v3.2.2 as a single partition and a phylogeny estimated under a GTR+I+G substitution model with no molecular clock imposed. Two independent runs (each with 4 chains) of 20 million generations were sampled every 2500 generations, with the tree topology estimated using the combined sample from the last 4 million generations of each run. Effective sample sizes of parameters within this sample were all >2000. A relaxed clock analysis was also performed in BEAST v2.1.3, using a GTR+I+G substitution model, relaxed log-normal clock and Yule speciation prior. This analysis used three independent runs of 30 million generations, sampled every 5000 generations, and the tree topology was estimated using the combined sample from the last 6 million generations of both runs. Effective sample sizes of parameters within this sample were all >100.

Whilst phylogenetic analysis can indicate how population-level samples are related within broader species-level sampling, inference of population level demographic histories within species requires application of methods that incorporate population-level processes, such as incomplete lineage sorting and gene flow, and explicitly estimate population demographic parameters. We assessed the utility of our dataset for population genomic inference using a recently developed likelihood-based approach (Lohse et al., 2011, 2012) designed for large numbers of sequences (i.e., sets of linked sites) rather than SNPs, to infer population divergence history. This method uses a coalescent framework to infer the relationship between a triplet of samples (here, a single individual from each of three *I. umbellifera* populations, repeated to allow different population combinations), and makes maximal use of data for minimal numbers of samples per population.

Here we provide the first application of this method to plant population data, inferring relationships among four regional populations of *I. umbellifera*, sampled in Panama, French Guiana, Peru, and Ecuador. We applied a modified version of Lohse et al.'s (2012) method that uses unrooted genealogies, and hence requires no outgroup. The method assumes that loci are unlinked with no intra-locus recombination. A key aspect of Lohse et al.'s (2012) method is that it allows assessment of support for population histories spanning the continuum from fully-resolved to wholly unstructured (a single panmictic population; **Figure 5A**). The completely resolved population model (see **Figure 5Ai**) estimates two population splitting times (*T*_1_ being the split between the two most recently diverged populations, *T*_2_ being the internode distance between the common ancestor of these two populations and the third population, measured in units of 2N_*e*_ generations) and the scaled mutation parameter θ (4N_*e*_μ). The likelihood framework of this method allows direct comparison of the support for a full model with a series of simpler nested models including a two-population model (**Figure 5Aii**, with *T*_1_ = 0), a polytomy model (**Figure 5Aiii**, with *T*_2_ = 0) and panmixis (**Figure 5Aiv**, with *T*_1_ = *T*_2_ = 0). We used the same 168 loci from the full accession dataset to infer the history of two population sets differing in geographic separation between populations: a maximal divergence set (Panama, French Guiana and Peru, with a pairwise separation of 2650–3000 km) and a minimal divergence alternative in which two western populations (Ecuador and Peru, separation 1485 km) are much closer to each other than either is to an eastern population (French Guiana, 2700–3000 km). For each set, analyses were repeated using a different individual from each population, following Hearn et al. (2014).

Power to discriminate among alternative scenarios derives primarily from the number of loci used in population genomic analyses, and bait capture technologies can deliver much larger numbers of loci than we use here. We therefore also present a power analysis to test how much targeted enrichment data with an average level of variation equivalent to that seen in the *Inga* data would be required to allow discrimination among the four nested population histories, for the maximal and minimal divergence population triplets. We calculated the expected difference in log likelihood between the full model and each of the nested models for a single locus. Since the loci are assumed to be unlinked, log likelihoods sum across loci enabling us to calculate the number of loci needed to achieve the likelihood difference required to reject a simpler model for a more complex one. We considered two levels of sequence variation by selecting two values of θ (2.1 or 1) that spanned the estimated values for our data (range = 0.99–1.48), and considered two timescales of population divergence, one very recent (*T*_1_ = 0.01 and *T*_2_ = 0.1) and one slightly older (*T*_1_ = 0.1 and *T*_2_ = 0.4).

## Results

### Transcriptomes

Assembly of the individual transcriptome libraries generated between 62,000 and 81,000 contigs >100 bp long per library with N50s of 1356–1836 bp (Table 1). The *Inga* reference transcriptome assembly used for selecting target loci contained 138,000 contigs with an N50 of 1615 bp. CEGMA results were similar across the single-species and combined transcriptomes, with all transcriptomes being almost complete (at least 94% of core eukaryotic genes present; Table 1). None of the CEGMA genes were single copy within *Inga* species (average copy number was greater than 2 for all transcriptomes), a pattern reflected in the initial read mappings, implying that a signature of genome duplication is clearly visible in the genus. Transcriptome assemblies and the bait design are available on the Dryad repository (Nicholls et al., 2015).

**Table 1:** **Assembly metrics for transcriptomes from three *Inga* species and the *Inga* reference transcriptome derived from the combined read data from all three accessions**.

	***I. umbellifera***	***I. spectabilis***	***I. sapindoides***	***Inga* reference**
Total length of reads (bp)	4,860,152,926	5,854,098,168	5,094,403,337	15,808,654,431
Length of assembled transcriptome (bp)	54,758,785	62,265,951	87,692,013	123,506,371
Total number of contigs	65,927	62,830	81,260	138,263
N50 length (bp)	1356	1668	1836	1615
N50 number	13,671	11,907	15,067	23,656
Maximum contig length (bp)	13,370	14,577	22,182	17,299
% GC	42.5	42.9	41.9	42.1
% of CEGMA proteins present as complete copies	94	97	97	96
Average number of complete orthologs per CEG	2.0	2.2	2.2	2.0
% of CEGMA proteins present as partial copies	97	99	97	99
Average number of partial orthologs per CEG	2.3	2.4	2.4	2.4

### Target loci sequences

Between 504,504 and 846,133 high quality paired-end reads were obtained from each genomic DNA library. Read data (for both genomic sequencing and the transcriptomes) are deposited at the European Nucleotide Archive (study ID ERP009747). The percentage of reads within a library that mapped back to the set of target loci was remarkably consistent, with nearly all libraries having between 71 and 80% of reads on target (range 61.9–80.7%; Table 2). The mean coverage of recovered target sequences ranged between 46 × and 91 × across libraries (Supplementary Table 4).

**Table 2:** **Read counts and percentage of reads that map to either the target locus set or the *Inga* plastid genome for each accession**.

**Accession**	**Species**	**Number of reads**	**% Reads matching baits**	**% Reads matching plastid DNA**
FG82	*I. alata*	736,094	77.66	5.98
FG156	*I. alba*	829,432	76.45	11.69
FG113	*I. auristellae*	772,882	76.01	6.70
FG185	*I. bourgonii*	678,681	77.21	6.75
FG_198	*I. brevipes*	668,910	71.68	8.71
FG_200	*I. brevipes*	661,645	73.07	9.59
KGD465	*I. cinnamomea*	812,966	74.81	7.75
FG35	*I. cylindrica*	798,644	77.77	6.93
FG192	*I. edulis*	808,684	76.34	6.53
KGD386	*I. edulis*	596,175	77.40	11.03
KGD345	*I. heterophylla*	782,120	80.33	8.92
FG89	*I. huberi*	777,591	77.54	8.86
KGD398	*I. laurina*	806,046	80.70	13.15
KGD355	*I. leiocalycina*	532,080	76.34	8.79
FG23	*I. longiflora*	704,139	77.01	7.94
FG83	*I. marginata*	741,408	78.66	6.47
FGIntype	*I. nouragensis*	717,932	75.02	6.09
FG21	*I. pezizifera*	749,113	79.89	5.84
KGD475	*I. punctata*	709,821	75.87	8.57
KGD388	*I. ruiziana*	709,968	78.53	7.93
BCI97	*I. sapindoides*	768,190	78.35	18.93
KGD343	*I. setosa*	632,574	77.41	8.47
FG94	*I. stipularis*	846,133	77.13	11.71
KGD110	*I. tenuistipula*	693,986	74.93	5.45
FG92	*I. thibaudiana*	750,949	77.05	8.00
BCI_103	*I. umbellifera*	705,519	72.08	11.74
FG_160	*I. umbellifera*	737,371	71.46	8.51
FG_180	*I. umbellifera*	695,691	72.99	9.25
FG_AN	*I. umbellifera*	640,046	71.17	9.01
FG_AP	*I. umbellifera*	648,202	71.37	7.57
FG_AR	*I. umbellifera*	594,861	73.20	9.54
FG_AZ	*I. umbellifera*	646,031	74.50	8.09
FG_AAA	*I. umbellifera*	531,997	73.58	9.95
FG_I	*I. umbellifera*	700,899	73.29	9.25
KD_401	*I. umbellifera*	689,439	74.43	9.18
KD_882	*I. umbellifera*	678,002	72.47	9.11
KD_882_replicate	*I. umbellifera*	504,504	61.93	11.06
KD_1059	*I. umbellifera*	624,086	72.73	8.64
KD_1316	*I. umbellifera*	618,017	70.93	7.71
TAKPDC_1272	*I. umbellifera*	665,173	76.01	9.07
TAKPDC_1318	*I. umbellifera*	544,312	78.45	11.36
TI_52	*I. umbellifera*	722,946	73.43	13.27
TI_908	*I. umbellifera*	691,496	71.89	10.57
TI_990	*I. umbellifera*	596,969	71.28	11.77
Yas_63659	*I. umbellifera*	618,876	72.72	10.04
Zygia917	*Zygia mediana*	680,243	72.65	10.29

**Figure 1:**
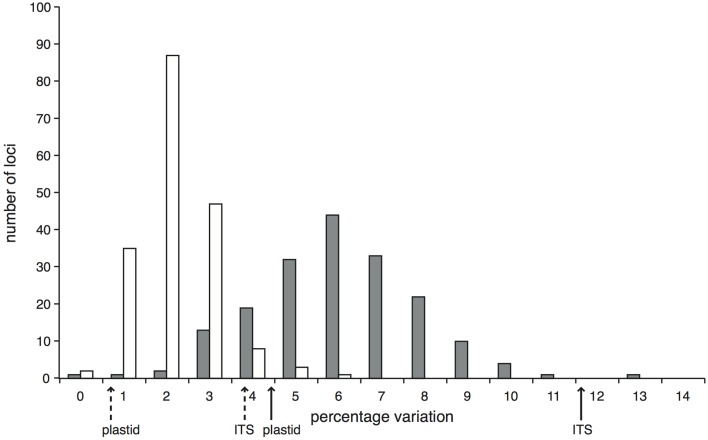
**Proportion of variable (gray bars) and parsimony informative (white bars) sites across the 183 *Inga* target loci enriched through hybrid capture that were selected for the phylogenetic analysis of 24 test accessions and that have data for at least three accessions**. Solid arrows below the x-axis indicate the percentage of variable sites within the Sanger-sequenced ITS and concatenated plastid loci; dashed arrows indicate the respective percentages of parsimony informative sites.

Within the comparison set of 24 phylogenetically-representative accessions, sequence data were recovered for every accession for the majority (87.1%) of target loci, with a further 4.2% of loci missing data from only a single accession (Supplementary Figure 5). The stringent mapping process and screening of loci resulted in 194 target loci in the final dataset. In 11 of these, data were only recovered for two or fewer accessions and so were not useful for phylogenetic reconstruction, giving 183 phylogenetically informative loci. We had an average of 180.5 (sd 1.3) loci per accession and 22.8 (sd 4.9) accessions per locus. The lengths of these informative alignments ranged between 194 and 4469 bp (mean 1707 bp, Supplementary Figure 6). The percentage of variable sites (including *Zygia*) ranged from 0 to 12.2% (mean 5.5%); the percentage of parsimony informative sites ranged from 0 to 5.0% (mean 1.7%; Figure 1). In comparison, the percentages of variable and parsimony informative sites in the Sanger sequence data were 4.9 and 1.2% for plastid DNA and 12.1 and 4.3% for ITS respectively (see Figure 1, Supplementary Table 3). This combination of variation levels and lengths provides enough data at each target locus to carry out multi-locus analyses that explore the data on a locus-by-locus basis (such as species tree reconstruction).

### Validation of mapping stringency and locus screening

Removal of paralogs would be expected to reduce “noise” contributing to long terminal branches. Both the stringent mapping of reads to the reference sequence and the removal of high coverage and outgroup-screened loci reduced the lengths of the terminal branches (Supplementary Figure 1). More stringent mapping had the greatest effect, with smaller reductions due to screening out loci. On average, terminal branches on the phylogeny based upon 183 stringently mapped, screened loci reduced to 48.7% of their length under default mapping values, with internal branches reducing only a small amount to an average of 91.5% of their initial length. The greatest reduction in terminal branch lengths was seen in the two *I. edulis* individuals, at 19.5 and 21.0% of their original lengths, with 248 variable sites between these two individuals across a total dataset of 245,474 shared sites (0.1%). Qualitatively similar results were obtained using more sophisticated Bayesian methods (implemented in BEAST), with minimal impact upon node support (data not shown).

### Species-level phylogenies from targeted enrichment data

Alignments for the comparison set of accessions across the 183 target loci remaining after screening contained a total of 315,188 bases, with 16,984 variable positions and 5220 parsimony informative sites. Once sites that had missing data for more than half the accessions were removed, the final concatenated alignment was 259,313 bp long, containing 16,433 (6.3%) variable sites and 4989 (1.9%) parsimony informative sites. Variation levels for the 60 loci selected for the species tree analysis ranged from 12.2 to 4.7% and 5.2 to 1.4% for variable and parsimony informative sites respectively (Supplementary Table 3). Final alignments are available on Dryad (Nicholls et al., 2015).

**Figure 2:**
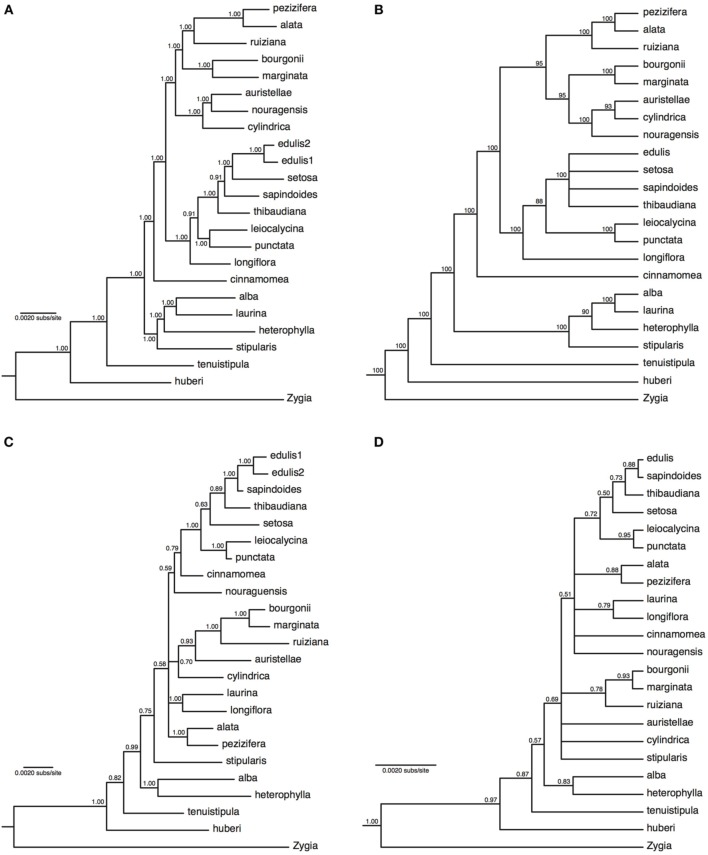
**Majority-rule consensus trees of 22 *Inga* species based on 183 nuclear loci obtained through targeted enrichment of genomic libraries and next-generation sequencing (A,B), or eight plastid genes and one nuclear gene (ITS) obtained through Sanger sequencing (C,D)**. **(A)** Analysis of concatenated next-generation data applying a single substitution model with no molecular clock; **(B)** majority-rule consensus cladogram of 22 *Inga* species based on a species tree analysis implemented in ASTRAL. Numbers next to nodes indicate bootstrap support; **(C)** analysis of concatenated Sanger data with gene-specific substitution models and no molecular clock; **(D)** species tree analysis of two loci (all plastid data and ITS) with locus-specific substitution models and relaxed clocks. Numbers next to nodes indicate posterior probability support.

The concatenated analyses produced identical and fully resolved tree topologies in relaxed clock and no-clock models, with posterior probabilities of one for all but two nodes in the no-clock MrBayes analysis (Figure 2A) and all but one node in the relaxed clock BEAST analysis (Supplementary Figure 7). Both types of species tree analyses also produced near-identical tree topologies that were almost fully resolved, albeit with lower support at some nodes (ASTRAL analysis in Figure 2B; ^*^BEAST analysis in Supplementary Figure 8). The species tree approach showed minor but well-supported topological differences within some of the major lineages relative to the concatenation approach. *Inga auristellea* is sister to *I. cylindrica* in the species tree analysis, but sister to *I. nouragensis* in the concatenated analysis. The species tree analysis also fails to resolve relationships among *I. edulis, I. setosa, I. sapindoides*, and *I. thibaudiana*. This degree of topological variation is to be expected between analyses with and without explicit incorporation of the gene tree discordance that is likely to be prevalent within the major lineages.

### Species-level phylogenies from plastid/ITS sequence data

Alignments of between 474 and 1381 bp were obtained from the eight plastid loci (with 5630 bp overall from plastid DNA) and 536 bp from ITS, with levels of variation ranging between 2.5 and 12.1% of sites (Supplementary Table 3). Data were obtained for most loci for most species, with 11% of locus by species combinations missing (Supplementary Table 2). Sequences are available in GenBank (accession numbers in Supplementary Table 2). The concatenated analyses (no-clock model from MrBayes in Figure 2C, relaxed clock model from BEAST in Supplementary Figure 9) and species tree analysis (Figure 2D) produced broadly congruent tree topologies; however, consistent with previously published *Inga* phylogenies there were multiple polytomies and poor support for many internal nodes. As expected for just two sampled loci with potentially incongruent histories, support in the species tree was on average lower than in the concatenated analysis (which was dominated by variation in plastid DNA). In both types of approach to analysing the data, the support for nodes was much lower than in the phylogenies derived from the much larger targeted enrichment dataset, with average posterior probability node support of 0.88 and 0.79 for the concatenated and species tree analyses respectively using the plastid/ITS data compared to 0.99 and 0.93 in the analyses of targeted enrichment data. It is also notable that the topology using the much larger targeted enrichment dataset is different to that obtained using the handful of previously sequenced loci. For example, *I. nouragensis* is sister to *I. auristellae* in the targeted enrichment dataset but sister to the *I. cinnamomea* and *I. edulis* clade in the smaller data set.

A large amount of plastid sequence was also recovered from each captured library (between 5.5 and 18.9% of reads; Table 2). We used the same stringent method to retrieve and assemble plastid data from the captured reads using as reference the complete plastid sequence of *Inga leiocalycina* (Dugas et al., in review), producing 5853 bp of aligned sequence. The short length of alignment is due to the high stringency of the pipeline and low coverage over much of the plastid genome. Most of the sequences assembled are regions matching or adjacent to three plastid-loci included in the full bait set (rbcL, cemA, and ccsA). Resolution in the resulting plastid phylogeny is poor and support for most branches very low, precluding any meaningful comparisons to the Sanger-sequenced plastid set or the full bait set (Supplementary Figure 10).

### Analyses of population-level divergence in *I. umbellifera*

**Figure 3:**
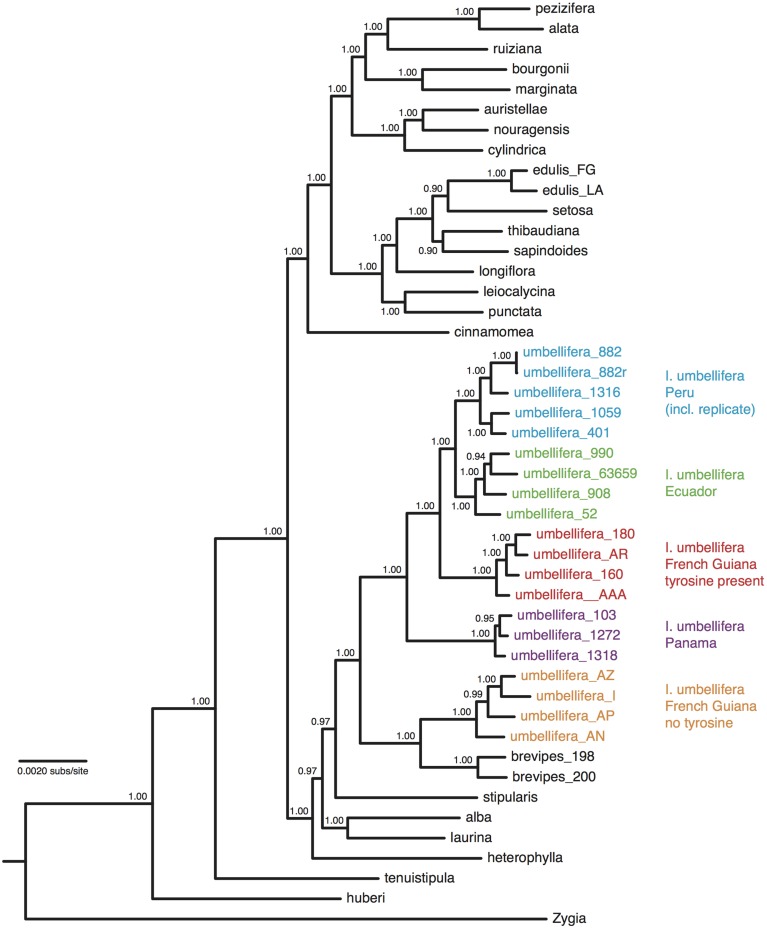
**Majority-rule consensus tree of 46 *Inga* accessions based upon Bayesian analysis of a concatenation of 168 nuclear loci obtained through targeted enrichment of genomic libraries and next-generation sequencing, applying a single substitution model with no assumption of a molecular clock**. Numbers next to nodes indicate posterior probability support. Geographic origins and chemotypes of *I. umbellifera* individuals are indicated, as is the technical replicate.

**Figure 4:**
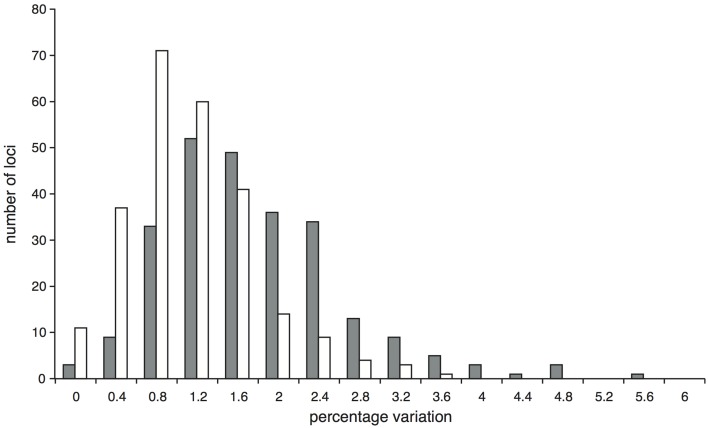
**Proportion of variable (gray bars) and parsimony informative (white bars) sites at the 251 *Inga* target loci enriched through hybrid capture for which data were obtained for at least two of the 19 *I. umbellifera* accessions**.

After trimming sites that had missing data for more than half the accessions, the concatenated alignment of 168 loci for the 22 accessions of population-level sampling within *I. umbellifera* and its close relative *I. brevipes* (including the technical replicate) plus the 24 comparison accessions contained a total of 224,786 bases, with 16,972 (7.6%) variable positions and 6345 (2.8%) parsimony informative sites. Analysis of these data using phylogenetic methods supported intra-specific divergence between *I. umbellifera* populations from different geographic areas, and also showed *I. brevipes* to be nested within *I. umbellifera* (Figure 3). Results also show that the two French Guianan *I. umbellifera* populations with distinct leaf chemistries, one with high levels of tyrosine, one without any tyrosine (Figure 3; Kursar pers. comm.), form separate, robustly supported clades which are not sister to each other. Both the no-clock and relaxed clock models gave identical topologies with almost identical support values. This topology was similar to that of the 24 accession analysis (Figure 2A), with a few minor though well-supported differences, which may reflect the extended taxon sampling. Variation per locus for the 19 *I. umbellifera* samples ranged from zero to 5.2% (mean 1.6%) and the proportion of parsimony informative sites ranged from zero to 3.5% (mean 0.9%; Figure 4). Variation between the two technical replicates of *I. umbellifera* sample KD882 was minimal, with only two sites differing out of the 209,239 sites in the final screened set of loci where data were present for both replicates, equivalent to an error rate of 0.00096%. This very low error rate suggests that the branch lengths we see for within-population parts of the phylogeny reflect true levels of variation, and that our approach limits the noise present in such large datasets.

The highly resolved differences between *I. umbellifera* populations shown in Figure 3 were obtained with methods that do not incorporate coalescent variation in gene tree topologies. Application of Lohse et al.'s (2012) method, which does so, reveals a much less resolved picture of population history, implying significant discordance between gene tree topologies at this taxonomic level in our data. In the maximal divergence set of populations, Panama was inferred to have diverged from the other two populations (*T*_2_ = 0.26, 95% *CI* = 0.14–0.38), but there was no signal in the data to distinguish the two-population vs. three-population models (i.e., we could not reject a value of 0 for *T*_1_, the divergence time between French Guiana and Peru). As might be expected, even less signal of population divergence was present in the minimal divergence population set (Peru, Ecuador, and French Guiana), even though phylogenetic methods support divergence between these three. Consistent with geographical proximity, the most divergent population was that from French Guiana (*T*_2_ = 0.15, consistent with the topology in Figure 3) although the 95% confidence interval for both this parameter and *T*_1_ (the divergence time between Peru and Ecuador) included zero.

**Figure 5:**
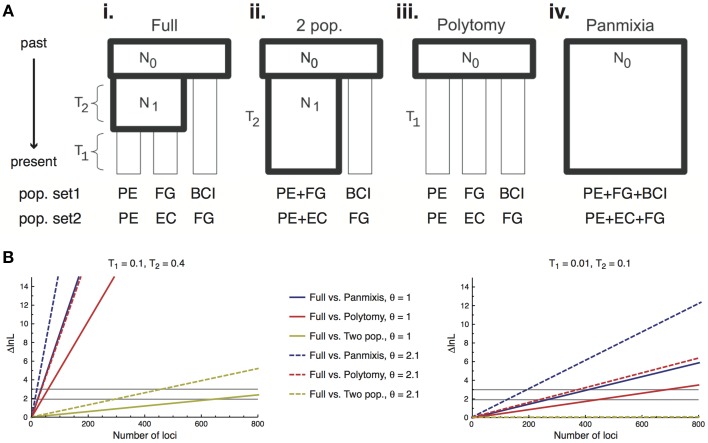
**(A)** Schematic of the four different population models tested using 168 loci obtained through targeted enrichment in *I. umbellifera*. Codes for populations in the two different test sets are: Panama, BCI; French Guiana, FG; Ecuador, EC; and Peru, PE. Diagram modified from (Lohse et al., 2012). **(B)** The expected difference in support (ΔlnL) between a full (three-population) model and each nested model as a function of the number of loci for older (left panel) and more recent (right panel) scenarios. The two horizontal black lines show the ΔlnL needed to reject the simpler model at α = 0.05 when the simpler model has one fewer parameter (polytomy and two-population models, lower line) or two fewer parameters (panmixis, upper line).

The power analyses showed fewer loci are required to resolve deeper nodes and older population histories. For the older divergence scenario (values of *T*_1_ = 0.1 and *T*_2_ = 0.4) data for fewer than 50 loci are required to resolve the older population split (*T*_2_) for either value of θ, and hence reject the two simplest unresolved population models (panmixis or a polytomy; Figure 5B, left panel). However, we would need data for almost 650/300 loci (for lower/higher θ values, respectively) to resolve the more recent population split (*T*_1_) and reject the two-population model in favor of the true three-population model. For the more recent divergence history (*T*_1_ = 0.01 and *T*_2_ = 0.1) even rejecting the simplest models in favor of a two-population model requires more loci than we currently have for *I. umbellifera*: we could confidently reject panmixis with 407/197 loci (lower/higher θ) or a polytomy model with 437/243 loci (lower/higher θ) (Figure 5B, right panel). We would need many thousands of loci to reject a two-population model in favor of the correct fully-resolved three-population model.

### Variation among different types of loci

We selected target loci using three different criteria: those we thought would be phylogenetically useful, those encoding genes differentially expressed between the three species used for transcriptomes and those encoding biosynthesis enzymes from secondary metabolic pathways. Differentially expressed target loci had lower levels of variation and those loci selected *a priori* to be phylogenetically useful had higher levels of variation [percentage of variable sites: ANOVA, *F*_(2, 180)_ = 5.23, *P* = 0.01; percentage of parsimony informative sites: ANOVA *F*_(2, 180)_ = 3.20, *P* = 0.04], although levels of variation among all three sets of genes were still relatively high (Supplementary Figure 11). To check whether this method of bait selection introduced biases which could affect species tree reconstruction, we also examined whether the different categories of loci produced different phylogenies. Concatenated alignments of sequences of each category were used to generate separate maximum likelihood phylogenies in RaxML (Supplementary Figures 12A–C). A fourth phylogeny was produced based upon a combined set of phylogenetically useful and differentially expressed loci containing the same number of loci as found in the set of secondary metabolism genes (Supplementary Figure 12D). Although there are some differences among the phylogenies they are minor and not well supported. Individual gene trees were also compared by category of bait and no patterns were discernable, although phylogenetic support based on any single locus was typically very low.

## Discussion

### Potential of targeted enrichment data

The genus *Inga* provides a robust test for the utility of targeted enrichment to resolve a recent, rapid and previously intractable radiation. Using targeted enrichment we have generated alignments of over 0.3 MB of coding sequence with 5220 informative sites, allowing complete resolution of a phylogeny for 24 accessions, compared to the poorly resolved phylogeny based upon Sanger-sequenced loci of the same species (Figure 2). Furthermore, the ability to resolve population relationships within *I. umbellifera*, and the power to discriminate among alternative population models (Figure 5) highlights another benefit of the targeted enrichment approach, namely simultaneous generation of data for inter- and intra-specific levels of analysis. The very low levels of variation seen in the technical replicate (less than one difference in 100,000 bp) give us confidence that this approach will provide robust characters for intra-specific studies. The bait set we used in this study is a subset of a larger set of 1400 loci designed for use across the legume subfamily Mimosoideae as a whole. Analysis using this larger set, comprising at least four times as many loci, will further enhance our understanding of the evolution of *Inga*, for instance by increasing our ability to discriminate among more parameter-rich models and/or more recent divergence histories.

Although more expensive than Sanger sequencing a small number of loci, the robustness of the phylogenies produced and the potential for economies of scale in increasing the number of accessions and loci makes this approach an attractive choice for phylogenetics. In addition, given the high species diversity of this genus and its young age (Pennington, 1997; Richardson et al., 2001) there is good reason to expect that this methodology, with similar numbers of loci, could resolve other recent plant radiations. Evolutionary radiations involve diversification across a continuum from populations to species, and data such as these also hold much promise for population genomic studies by providing the large numbers of loci required for discrimination among competing population history models (Lohse et al., 2011). Furthermore, more in-depth analysis of data such as these can be used for much more than simply reconstructing population histories or phylogenies, with potential to address a range of issues such as assessing levels of introgression among species, the prevalence and nature of gene flow during speciation and the role of polyploidization in speciation (Arnold et al., 2015).

The species-tree methods employed here explicitly allow for differing coalescent histories and mutation models among loci (Maddison, 1997; Edwards, 2009) unlike the concatenation approach. However, the congruence between the concatenation-based phylogeny, and the species-tree based phylogeny, is striking. This suggests there is strong signal in the data to reveal the history of this radiation. There are some topological differences between phylogenies derived using species tree vs. concatenation approaches but these are found toward the tips of the phylogenies—exactly where we would expect maximum contrast between methods that do and do not incorporate coalescent variation in gene trees as concatenation can yield well-supported but incorrect topologies in the presence of significant incomplete lineage sorting (Kubatko and Degnan, 2007; Salichos and Rokas, 2013; Roch and Steel, 2014). In addition, terminal branch lengths were generally shorter in the species tree, which again may be expected since multi-species coalescent models may place gene coalescences prior to speciation events. With genomic-scale datasets, the choice of substitution or heterogeneity model can also have a large impact on the support for particular topologies (Kumar et al., 2012; Wickett et al., 2014). However, the benefits of a large dataset are clear, as the comparison with the plastid/ITS data shows that with only a few loci the tree topology obtained (irrespective of analysis models) is likely to be a biased estimate of the true species tree.

### Advantages over other next-generation sequencing methods

An alternative reduced-representation sequencing method, RAD sequencing, allows generation of large numbers of markers for many accessions without any initial genomic or transcriptomic information and at significantly lower cost (Davey et al., 2011; see Eaton and Ree, 2013 for an application to phylogenetics). Although targeted enrichment does require some initial sequence information, it has several advantages over RAD-based approaches. The loci used are known sequences, so choice of targets can be focused on particular levels of variation or specific genetic/metabolic pathways of interest, and hence can be used in wider functional phylogenomic analyses, including gene family evolution or patterns of molecular evolution. The small differences seen in the phylogenetic utility of the three types of baits we used suggests that hybrid capture experiments could be designed to allow such multipurpose use of the data. In addition, the long and variable alignments which can be assembled for each locus allow for more flexible and powerful phylogenetic and population-level analyses than is possible from either concatenation of SNPs from anonymous sequences (McCormack et al., 2013) or short sequences obtained via RAD sequencing (Eaton and Ree, 2013). Finally, the fact that selection of loci is intrinsic to the design of a targeted enrichment sequencing experiment means that obtaining orthologous data with appropriate levels of variation across all samples is more certain, something not always achievable using low-coverage genomic skimming (Hearn et al., 2014).

Our bioinformatics pipeline was designed to generate consensus sequences for phylogenetic inference, particularly to avoid retention of paralogous loci. As such, heterozygous sites in individual samples were coded as Ns so are uninformative for analysis. At the population genetic level, this action removes potentially informative sites and means that our divergence time and effective population size estimates may be underestimates. Ideally, one would have phase information (e.g., from back mapping to a robust reference genome or via random phasing) so that these sites can be retained in the analysis and we recommend this approach in future studies focused on population level analyses. Nevertheless, our analysis of *I. umbellifera* populations demonstrates that even our current dataset can differentiate relative divergence times of nearby and distant populations.

### *Inga* phylogenetics

There are four papers containing phylogenetic hypotheses for *Inga* (Richardson et al., 2001; Lavin, 2006; Kursar et al., 2009; Dexter et al., 2010). Richardson et al. (2001) analyzed ITS and plastid *trnLF* sequences for a total of 45 *Inga* species from across the range of the genus, and Lavin (2006) reanalysed the same ITS dataset. Kursar et al. used more sequence data—six plastid regions—for 37 *Inga* species from Panama and Peru. Dexter et al. (2010) analyzed more species than the earlier studies (55) from a relatively small area of Amazonian Peru, using ITS and the plastid *trnDT* region.

The hallmark of all these published phylogenies is a backbone that is entirely unresolved (e.g., by ITS and *trnL* alone; Richardson et al., 2001) or at best poorly resolved, with numerous polytomies or nodes that receive little support from Bayesian posterior probabilities or bootstrap values (Kursar et al., 2009; Dexter et al., 2010). The phylogenies presented here based upon targeted enrichment data are a major improvement because they resolve all nodes in the tree, especially more basal ones, with high support—for the first time a well-resolved phylogenetic backbone is available for the genus. This has considerable implications for both corroborating past evolutionary studies of the genus and for future studies of its biology and classification.

For example, the now well-supported order of relationships, which is not congruent with their chemical similarities as determined by Kursar et al. (2009), confirms the conclusion that closely related species do not have similar defense chemistry, which contradicts a widely held assumption in co-evolutionary theory that closely related species should be chemically similar (Ehrlich and Raven, 1964). For example, *I. edulis* and *I. tenuistipula*, shown to be distantly related here (Figures 2A,B) are chemically similar (Kursar et al., 2009). The new, well-supported phylogenetic hypothesis also confirms the suspicion based on the prior, weakly resolved phylogenies, that the taxonomic sections in the genus (Pennington, 1997) are non-monophyletic and will require re-circumscription. For example, species sampled here from sect. Leptinga (*I. tenuistipula, I. huberi, I. brevipes, I. umbellifera, I. cinnamomea*) are not resolved as a monophyletic group. The non-monophyly of these sections implies that morphological character evolution in *Inga* requires re-evalution, and this could be done in the context of a phylogeny sampling more species. Even in the absence of thorough species sampling, some intriguing hints emerge. For example, all the species in the clade that includes species from *I. longiflora* to *I. edulis* have conspicuous orange to reddish brown hairs on the twigs, rachises and leaflet undersurfaces. The apparent conservatism in this pubescence character may relate to the function of these hairs in deterring insect herbivores, which is a potential defense of *Inga* species not examined by Kursar et al. (2009).

### Methodological issues

Other studies (e.g., Mandel et al., 2014; Weitemier et al., 2014; Grover et al., 2015) have focused on finding single copy genes for targeted enrichment using reference genomes from species within their study taxon, but without a reference *Inga* genome we cannot check directly for copy number in our target loci. In addition, knowledge of ploidy levels in *Inga* is far from complete but the limited data available suggest around 11% of species may be polyploid and ploidy may be variable within species (Hanson, 1997; Figueiredo et al., 2014). Yet the presence of paralogs within all *Inga* and *Zygia* accessions sequenced in this study indicates that the enrichment technique is sensitive enough to pick up the signature of an older whole genome duplication recently hypothesized to have occurred at the base of the Mimosoideae-Caesalpinieae-Cassieae (MCC) clade containing subfamily Mimosoideae, to which *Inga* belongs (Cannon et al., 2015). If not accounted for, polyploidy and genome duplications can introduce considerable paralogy issues into phylogenomic datasets, potentially misleading phylogenetic inference (Lemmon and Lemmon, 2013). However, the empirically-determined stringent mapping parameters and locus screening steps employed here show that it is possible to recover sequences likely to be orthologous across accessions even when we have limited starting information about target loci.

Several other targeted enrichment studies have used *de novo* assembly in addition to mapping to derive phylogenetically useful sequences (Weitemier et al., 2014; Grover et al., 2015; Heyduk et al., 2015; Stephens et al., 2015a,b). With sufficient coverage, *de novo* assembly provides access to the sometimes significant proportion of the sequence data in targeted sequencing experiments which does not map directly to the target loci, instead coming from regions adjacent to baits and from high-copy number loci such as those from organelles. However, in lineages with a history of gene duplications paralogs may be co-enriched by a single bait, as appears to be the case in *Inga*. These paralogous sequences make *de novo* assembly difficult. A mapping-only approach has some advantages in its simplicity, speed, and utility for phylogenetic reconstruction. In addition, a mapping approach allowed us to inspect read alignments by eye to confirm that increases in stringency were having the desired effect at removing paralogs, hence providing a quality control step at an intermediate stage between quality-trimmed reads and the final consensus sequence. We have used conservative parameters to exclude paralogs during mapping but with stringent enough assembly parameters and sufficient coverage of all members of a gene family, one could potentially assemble and then discriminate among the different paralogs in each accession using gene tree-based orthology approaches (Yang and Smith, 2014).

Targeted enrichment generates much more data than Sanger sequencing but is this worth the increased cost and technical difficulties? Most lab budgets can withstand a few failed PCRs and Sanger sequencing reactions. In contrast, the construction of next-generation sequencing libraries and the hybrid capture process are technologically more challenging and are expensive if they fail. Despite this, we consider that for anything above a small-scale project this technology should be considered because the cost per base pair is vastly lower, and careful balancing of the number of baits, the multiplexing of libraries within hybridization reactions and sequencing effort can ensure that little of the large volume of extra data is wasted. In addition, by virtue of the enrichment process, with appropriate selection of the numbers and types of loci there is greater guarantee of sufficient depth of sequencing to resolve a phylogeny or differentiate population models. However, the mistaken inclusion of three plastid loci in the wider bait set confirms the obvious disadvantages of combining organelle and nuclear sequences in a single capture reaction due to the high number of reads mapping to the plastid baits resulting in waste of sequencing efforts. The same argument militates against inclusion of repetitive sequences and high copy number genes in nuclear bait sets.

The quality of the DNA required for next-generation libraries is also a factor to be considered. PCR amplicons, in particular when amplifying multi-copy loci such as plastid sequence and ITS, can be obtained from poor quality DNA samples. However, the production of a good next-generation sequencing library from poor quality genomic DNA is more difficult. A number of library-prep kits are available to deal with this issue by using small amounts of the best quality genomic DNA extractable (down to 10 pg), or optimizing them for denatured and nicked DNA. However, their compatibility with the hybrid capture system should be confirmed as we have found strong variation in the success of this approach between library kits; for example target loci within libraries of *Inga* DNA made using Illumina's Nextera kits did not enrich under the same protocol as described above. As good quality libraries can be prepared even from old herbarium material, targeted enrichment offers a method to access large amounts of sequence data from these specimens, opening up collections to large scale genetic analysis (Bi et al., 2013; Carpenter et al., 2013; Samuels et al., 2013; Staats et al., 2013; Besnard et al., 2014).

## Conclusion

We have demonstrated the utility of targeted enrichment of nuclear gene sequences for resolving phylogenies of recent plant radiations. The work described here is part of a project that will eventually include a much larger number of species, with multiple accessions per species and larger numbers of genes. The intra-specific population genomic power analysis for the 19 accessions of the widespread species *I. umbellifera* suggests this method can also generate useful within-species phylogeographic markers, an idea supported by the use of this method for SNP-based functional genetic studies (Salmon et al., 2012; Winfield et al., 2012; Zhou and Holliday, 2012; Tennessen et al., 2013). The robust resolution observed in this system is based upon data from exonic regions only, but with additional genomic information baits could be designed to target more variable regions of the genome, further improving the potential resolving power at species-complex and population levels, for instance allowing resolution among models of population divergence with and without gene flow, or addressing questions of speciation through hybridization. The relatively uniform enrichment and coverage we describe here is repeatable over five separate hybridizations, even with varying quality of input genomic DNA. We hope to be able to extend this methodology to DNA extracted from herbarium specimens, greatly expanding the general utility of this approach for plant phylogenetics.

### Conflict of interest statement

The authors declare that the research was conducted in the absence of any commercial or financial relationships that could be construed as a potential conflict of interest.

## Supplementary material

Supplementary Figure 1 **Effect of greater stringency in read mapping, variant calling and screening of loci containing paralogs on branch lengths and topology of neighbor-joining trees from analyses of concatenated loci**. **(A)** Default Bowtie2 mapping parameters (275 loci in total); **(B)** stringent mapping parameters, all loci (264 loci); **(C)** stringent mapping parameters, excluding loci with high coverage (248 loci); and **(D)** stringent mapping parameters, excluding loci with high coverage and loci where removing outgroup makes minimal difference to variation (194 loci).

Supplementary Figure 2 **Change in the percentage of sites called as different from the reference and the change in the standardized quality of those variant sites in response to increasing the constant value when calculating the alignment score threshold in Bowtie2**. Data are for three *Inga* accessions: **top row**, *I. sapindoides* BCI97; **middle row**, *I. alata* FG82; **bottom row**, *I. alba* FG156.

Supplementary Figure 3 **The number of bases excluded in response to increasing the minimum quality score required for retention in the vcf file from 24 to 48, in increments of 3, assessed for the total number of bases (top panel) and the number of variant calls (bottom panel)**.

Supplementary Figure 4 **Percentage change in the proportion of variable sites within the 252 variable loci containing the 24 test accessions calculated when including and excluding the outgroup taxon *Zygia***.

Supplementary Figure 5 **Frequency of different numbers of missing accessions for *Inga* target loci assembled by the pipeline**.

Supplementary Figure 6 **Distribution of alignment lengths across the 183 *Inga* target loci enriched through hybrid capture that were selected for the phylogenetic analysis of 24 test accessions and that have data for at least three accessions**.

Supplementary Figure 7 **Majority-rule consensus tree of 22 *Inga* species based on Bayesian analysis of 194 concatenated nuclear loci obtained through targeted enrichment of genomic libraries and next-generation sequencing, analyzed using a single substitution model and relaxed clock**. Numbers next to nodes indicate posterior probability support.

Supplementary Figure 8 **Majority-rule consensus species tree analysis of 22 *Inga* species based on Bayesian analyses of sequences from 60 nuclear loci with locus-specific substitution models and relaxed clocks**. Numbers next to nodes indicate posterior probability support.

Supplementary Figure 9 **Majority-rule consensus tree of 22 *Inga* species based on Bayesian analyses of a concatenation of eight plastid genes and one nuclear gene (ITS) obtained through Sanger sequencing, applying gene-specific substitution models and relaxed molecular clocks**. Numbers next to nodes indicate posterior probability support.

Supplementary Figure 10 **Majority-rule consensus tree of 22 *Inga* species based on Bayesian analyses of plastid loci obtained as a by-product of targeted enrichment of genomic libraries and next-generation sequencing, analyzed using a single substitution model and no molecular clock**. Numbers next to nodes indicate posterior probability support.

Supplementary Figure 11 **Mean percentage (±SE) of variable (gray bars) and parsimony informative sites (white bars) across the three types of target loci**.

Supplementary Figure 12 **Maximum likelihood phylogenies derived using the three different types of target loci**. **(A)** Phylogenetically informative loci (30 loci, 45,648 bp); **(B)** differentially expressed loci (72 loci, 119,011 bp); **(C)** secondary synthesis pathway loci (66 loci, 111,455 bp); and **(D)** non-secondary synthesis loci (66 loci, 133,754 bp) consisting of 30 phylogenetically informative loci and 36 differentially expressed loci. Numbers next to nodes indicate bootstrap support.

Supplementary Table 1 **Annotation of the *Inga* loci selected for targeted enrichment**.

Supplementary Table 2 **Collection details and information on inclusion of different *Inga* accessions in the transcriptome, targeted enrichment and Sanger sequencing data sets**. GenBank accession numbers are presented for each gene by species combination that was Sanger sequenced.

Supplementary Table 3 **Details of sequence variation and substitution models for the Sanger-sequenced and targeted enrichment loci**.

Supplementary Table 4 **Coverage for each target locus within each accession**. Coverage per locus was calculated from the read mappings by summing the coverage at each base then dividing by the length of the target locus sequence from the reference transcriptome.
