# Supplementary figures and images for: Using targeted enrichment of nuclear genes to increase phylogenetic resolution in the neotropical rain forest genus Inga (Leguminosae: Mimosoideae)
Source: Front Plant Sci. 2015 Sep 17;6:710. doi: 10.3389/fpls.2015.00710 (PMC4584976; doi:10.3389/fpls.2015.00710)

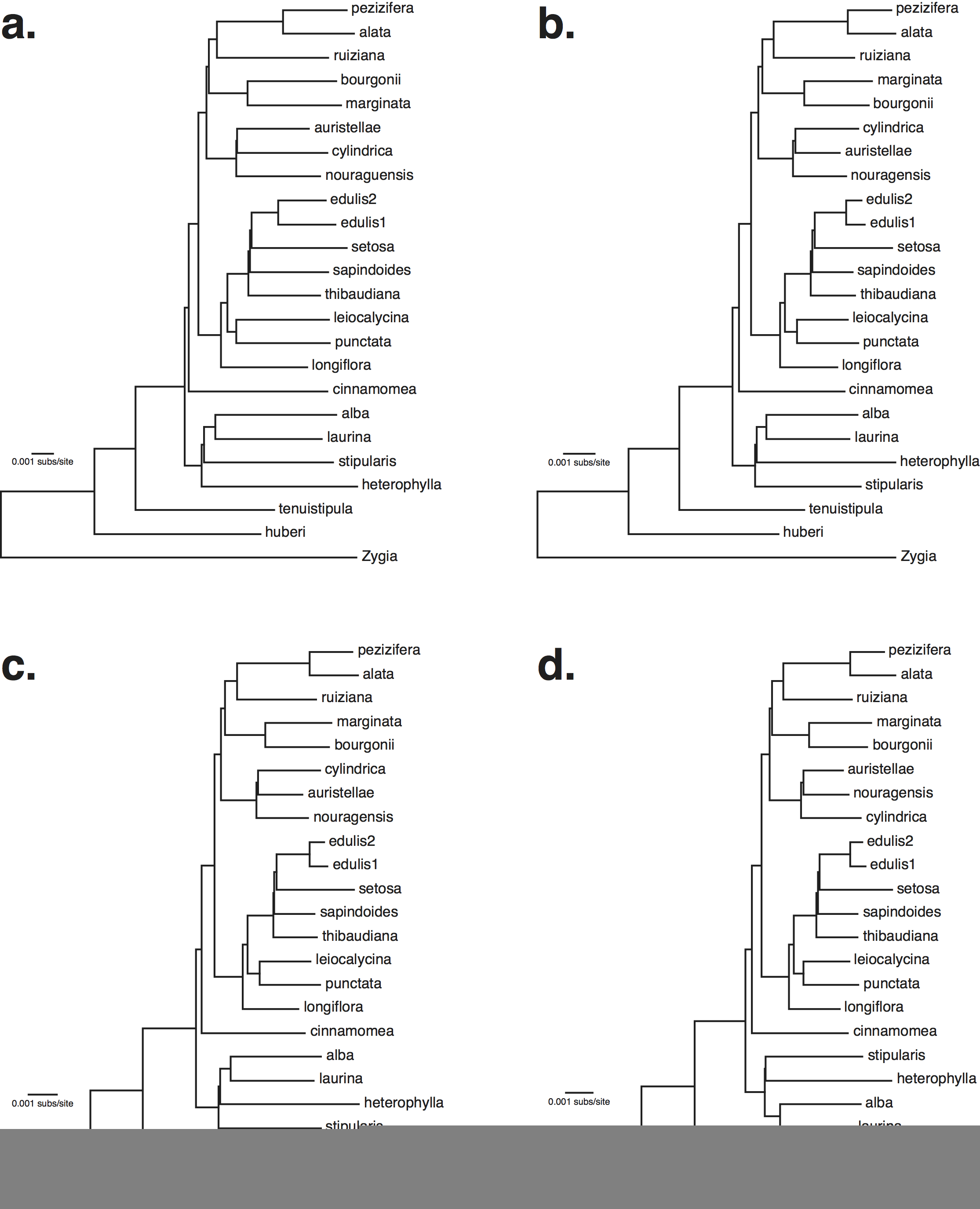

Supplement: Supplementary Figure 1 — Effect of greater stringency in read mapping, variant calling and screening of loci containing paralogs on branch lengths and topology of neighbor-joining trees from analyses of concatenated loci. (A) Default Bowtie2 mapping parameters (275 loci in total); (B) stringent mapping parameters, all loci (264 loci); (C) stringent mapping parameters, excluding loci with high coverage (248 loci); and (D) stringent mapping parameters, excluding loci with high coverage and loci where removing outgroup makes minimal difference to variation (194 loci). [file Image1.JPEG]

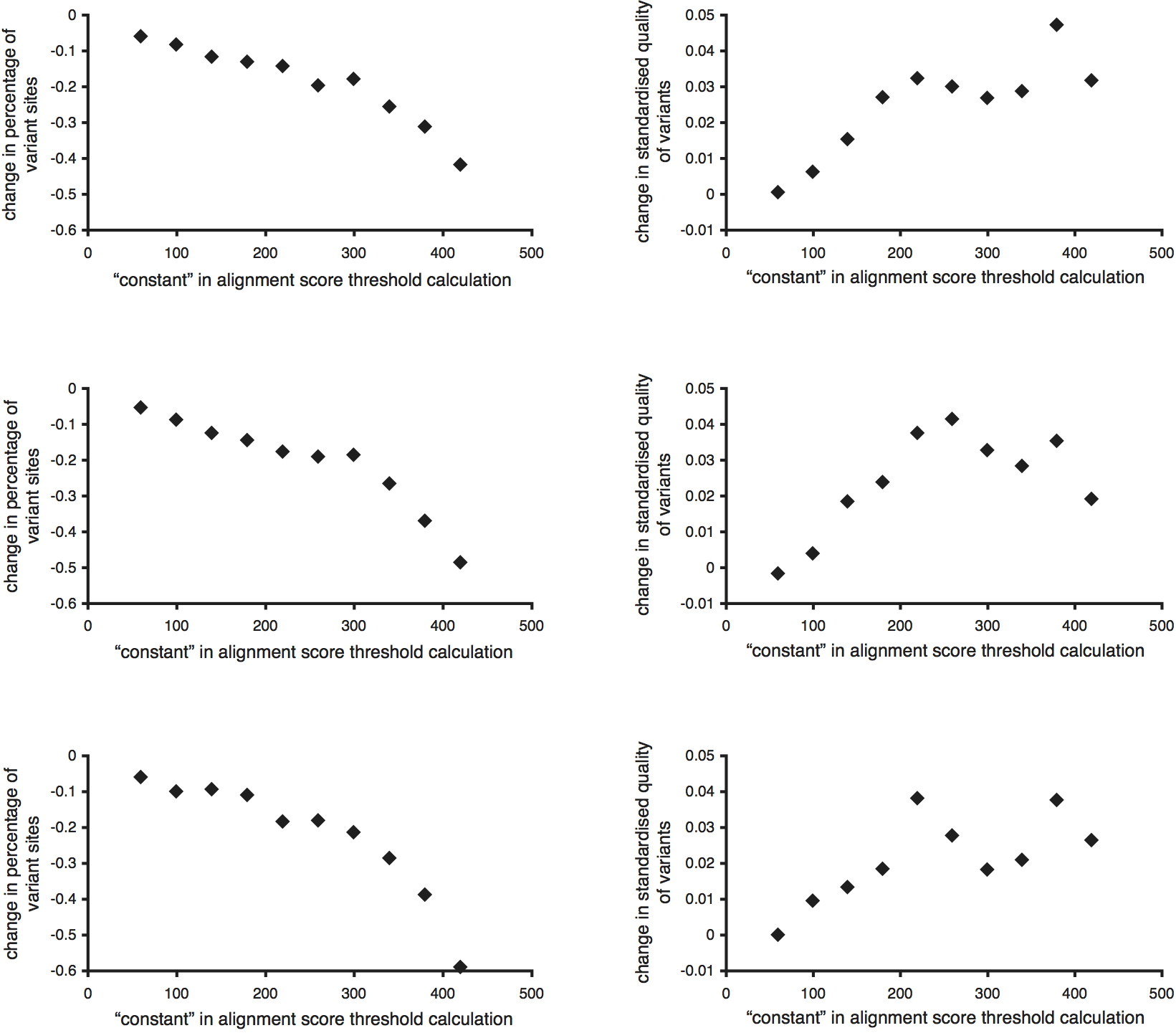

Supplement: Supplementary Figure 2 — Change in the percentage of sites called as different from the reference and the change in the standardized quality of those variant sites in response to increasing the constant value when calculating the alignment score threshold in Bowtie2. Data are for three Inga accessions: top row, I. sapindoides BCI97; middle row, I. alata FG82; bottom row, I. alba FG156. [file Image2.JPEG]

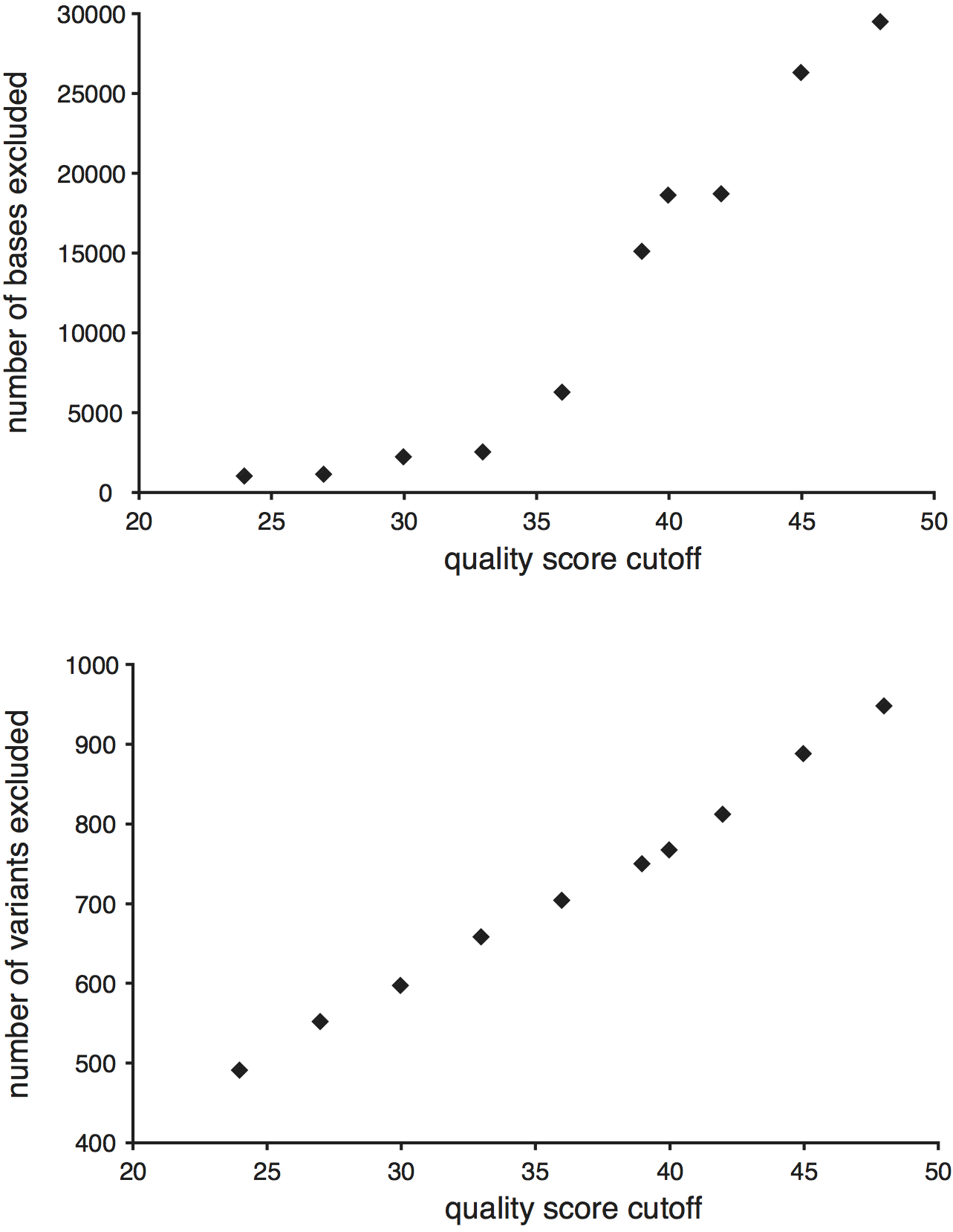

Supplement: Supplementary Figure 3 — The number of bases excluded in response to increasing the minimum quality score required for retention in the vcf file from 24 to 48, in increments of 3, assessed for the total number of bases (top panel) and the number of variant calls (bottom panel). [file Image3.JPEG]

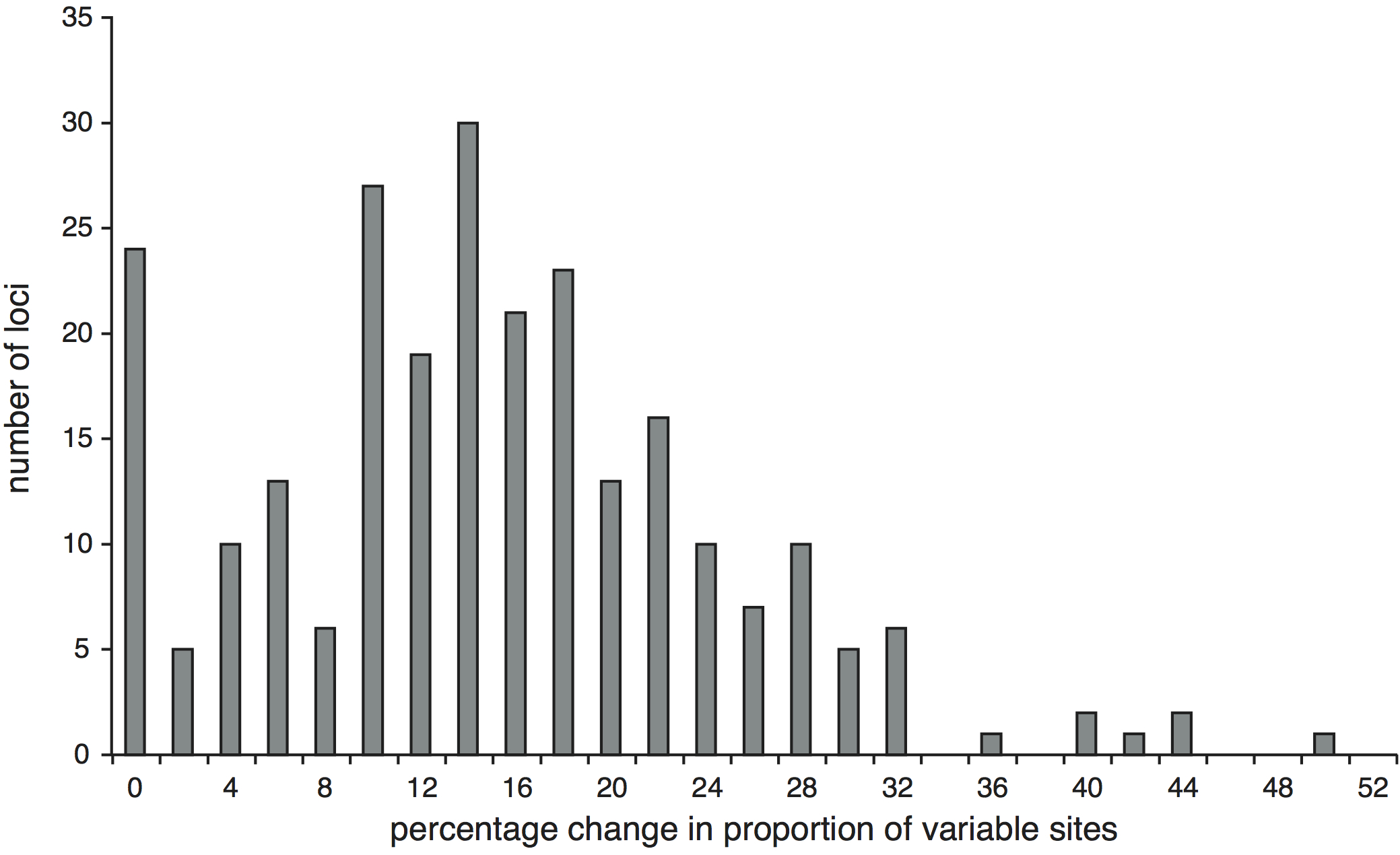

Supplement: Supplementary Figure 4 — Percentage change in the proportion of variable sites within the 252 variable loci containing the 24 test accessions calculated when including and excluding the outgroup taxon Zygia. [file Image4.JPEG]

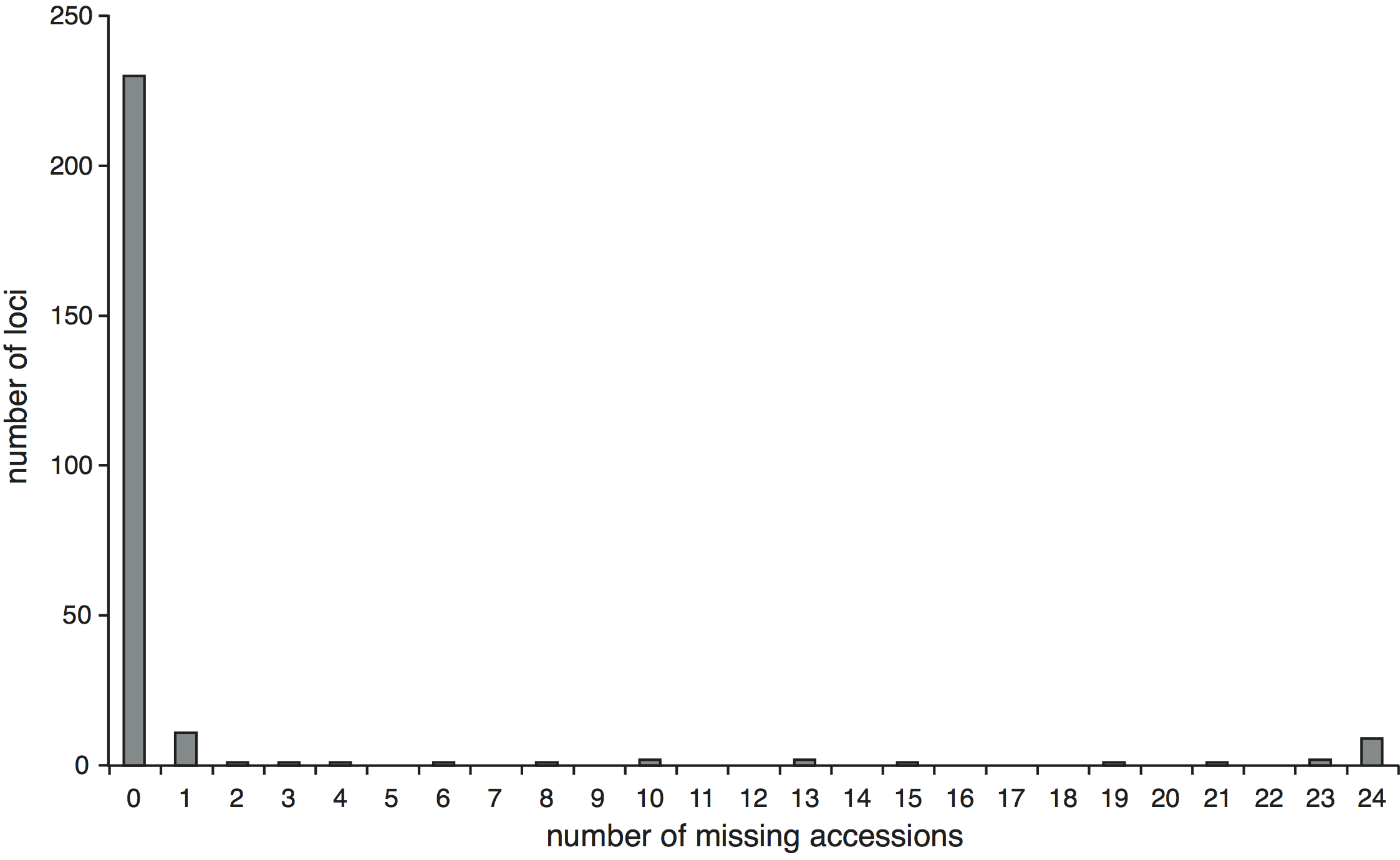

Supplement: Supplementary Figure 5 — Frequency of different numbers of missing accessions for Inga target loci assembled by the pipeline. [file Image5.JPEG]

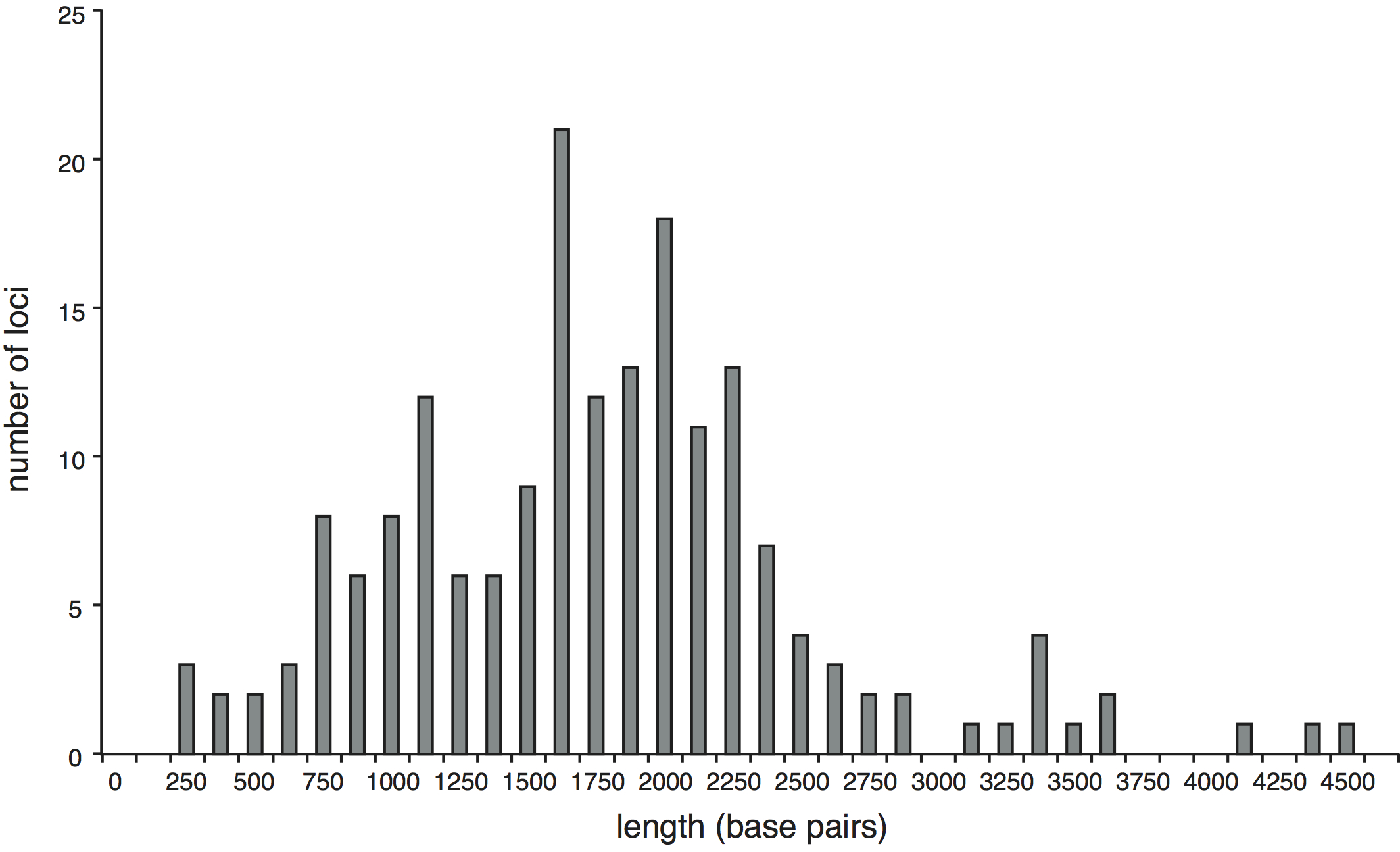

Supplement: Supplementary Figure 6 — Distribution of alignment lengths across the 183 Inga target loci enriched through hybrid capture that were selected for the phylogenetic analysis of 24 test accessions and that have data for at least three accessions. [file Image6.JPEG]

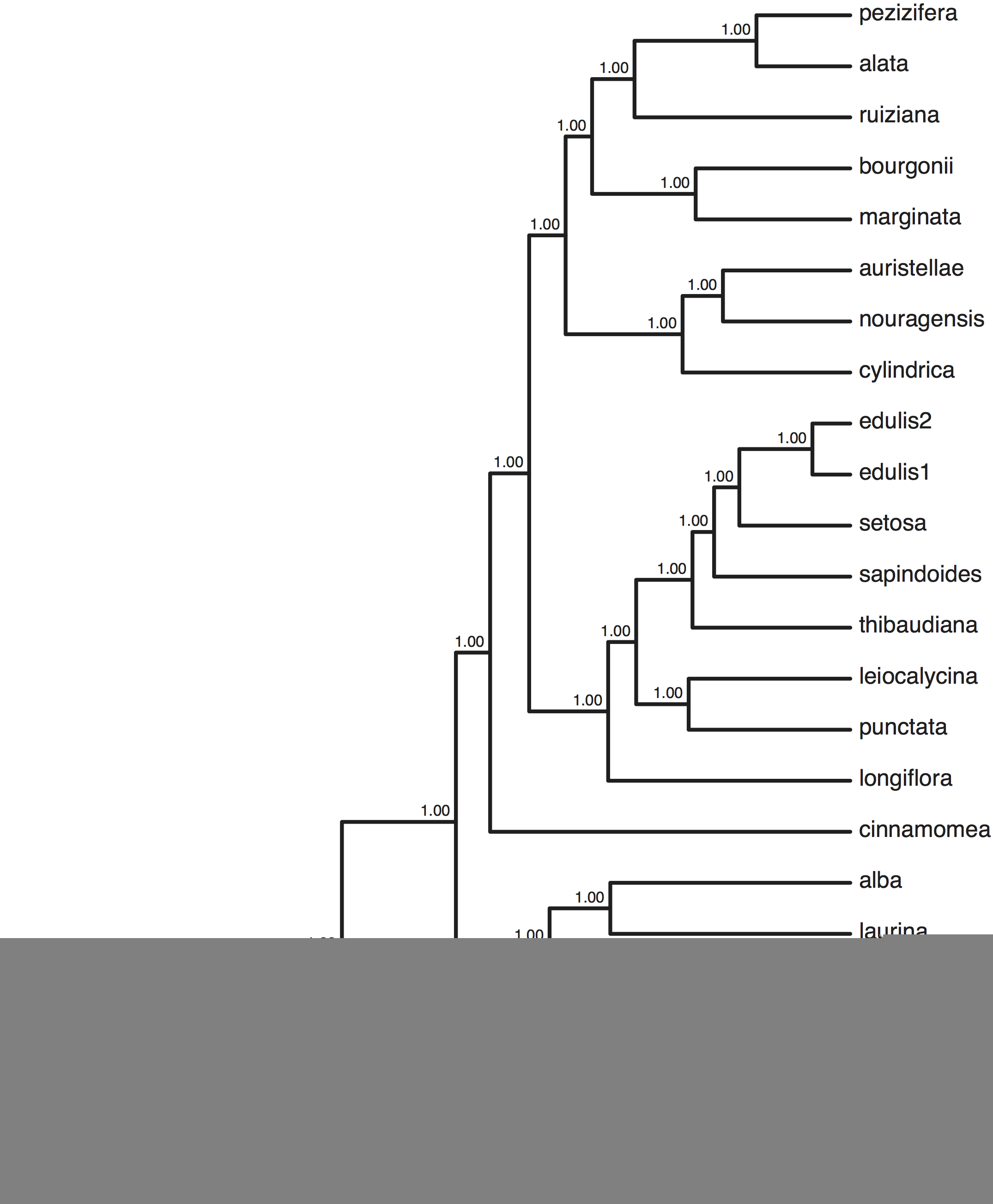

Supplement: Supplementary Figure 7 — Majority-rule consensus tree of 22 Inga species based on Bayesian analysis of 194 concatenated nuclear loci obtained through targeted enrichment of genomic libraries and next-generation sequencing, analyzed using a single substitution model and relaxed clock. Numbers next to nodes indicate posterior probability support. [file Image7.JPEG]

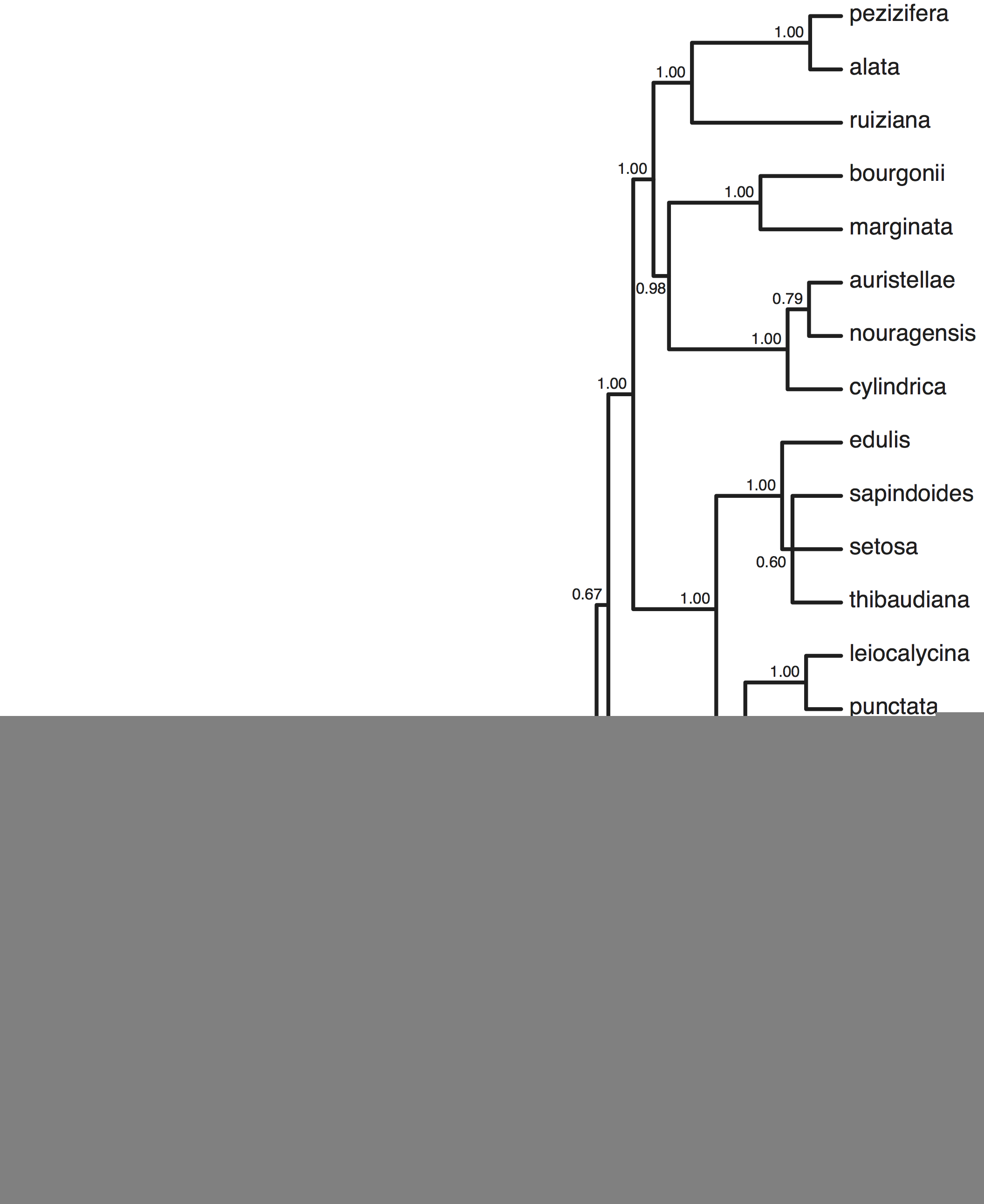

Supplement: Supplementary Figure 8 — Majority-rule consensus species tree analysis of 22 Inga species based on Bayesian analyses of sequences from 60 nuclear loci with locus-specific substitution models and relaxed clocks. Numbers next to nodes indicate posterior probability support. [file Image8.JPEG]

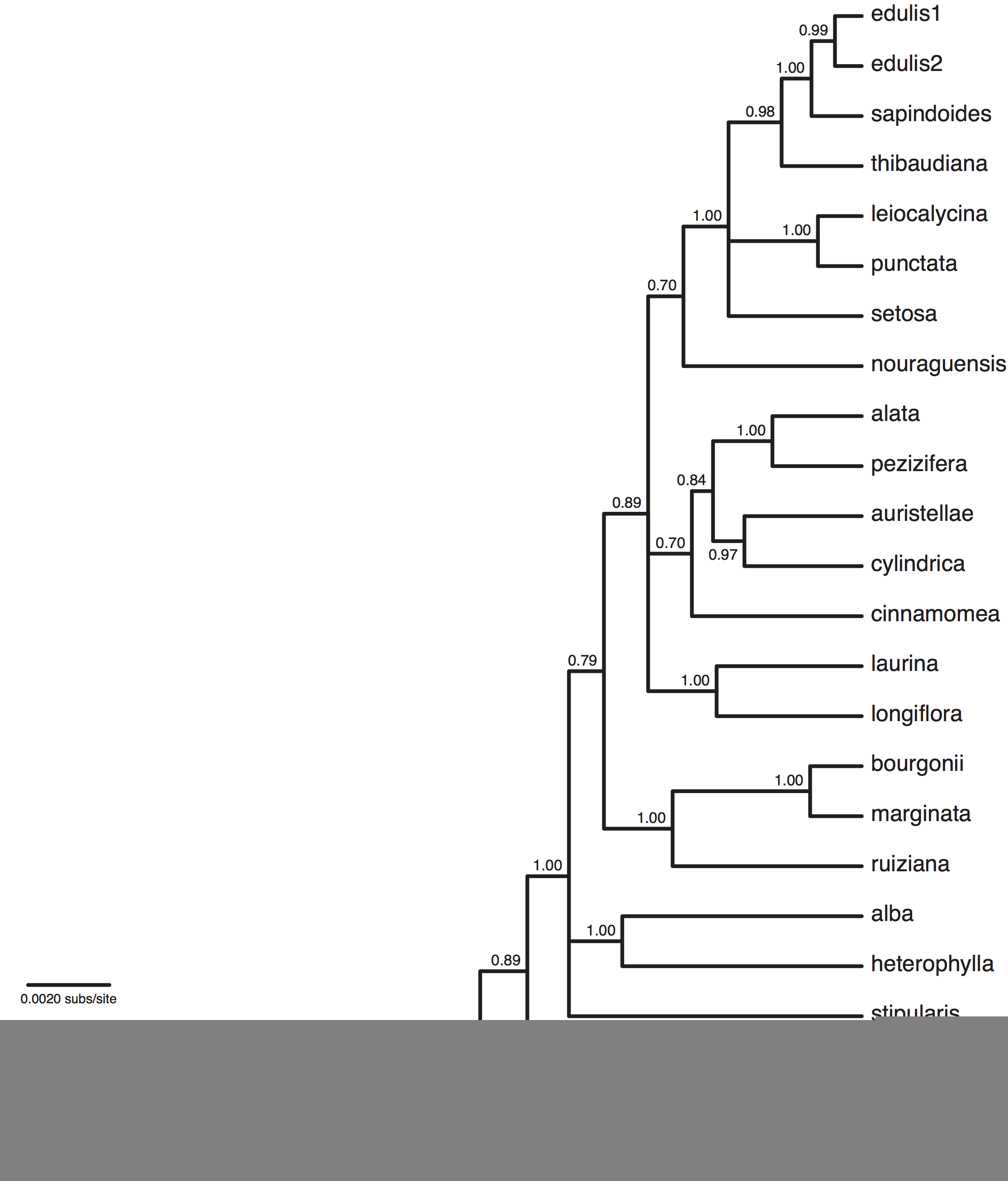

Supplement: Supplementary Figure 9 — Majority-rule consensus tree of 22 Inga species based on Bayesian analyses of a concatenation of eight plastid genes and one nuclear gene (ITS) obtained through Sanger sequencing, applying gene-specific substitution models and relaxed molecular clocks. Numbers next to nodes indicate posterior probability support. [file Image9.JPEG]

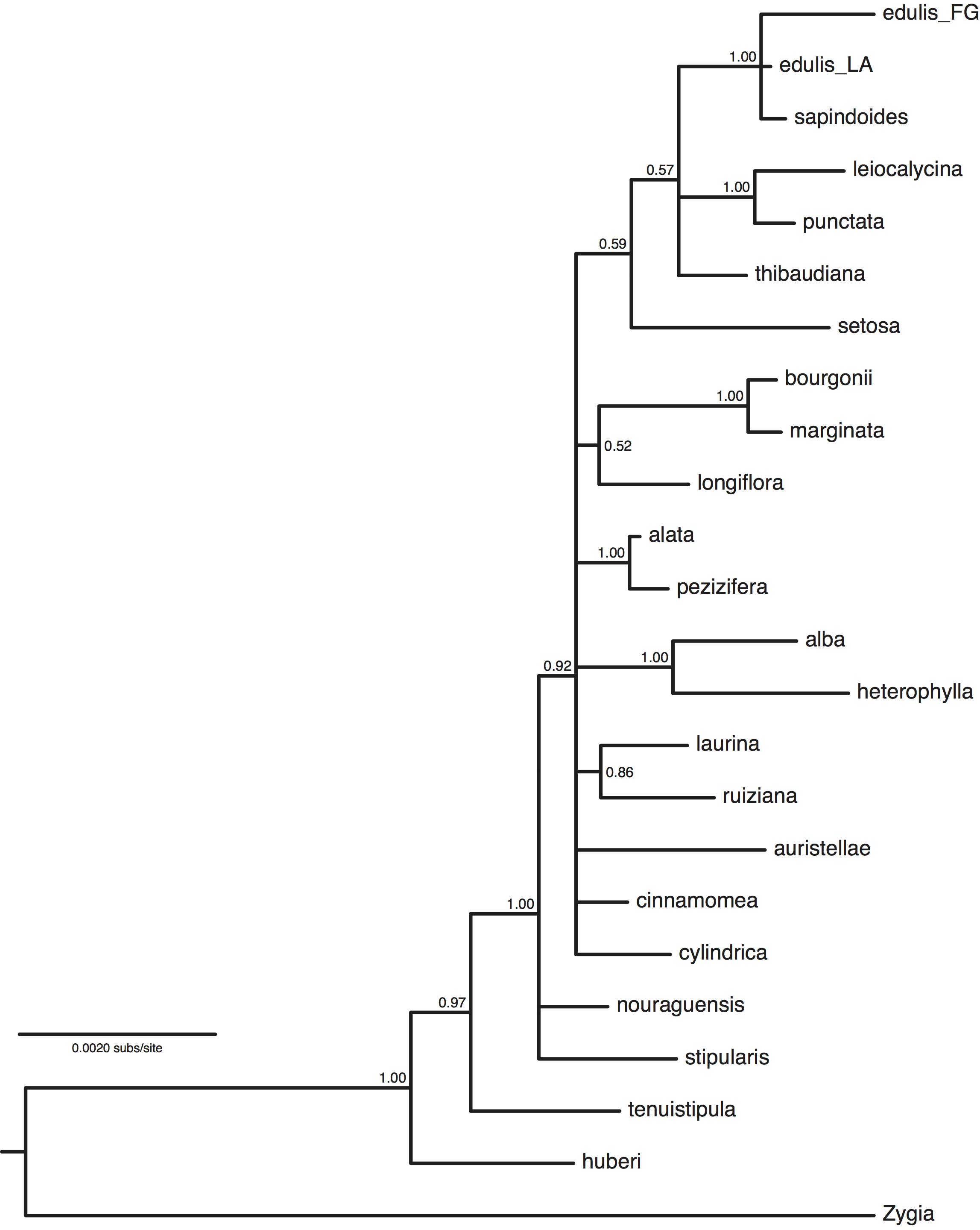

Supplement: Supplementary Figure 10 — Majority-rule consensus tree of 22 Inga species based on Bayesian analyses of plastid loci obtained as a by-product of targeted enrichment of genomic libraries and next-generation sequencing, analyzed using a single substitution model and no molecular clock. Numbers next to nodes indicate posterior probability support. [file Image10.JPEG]

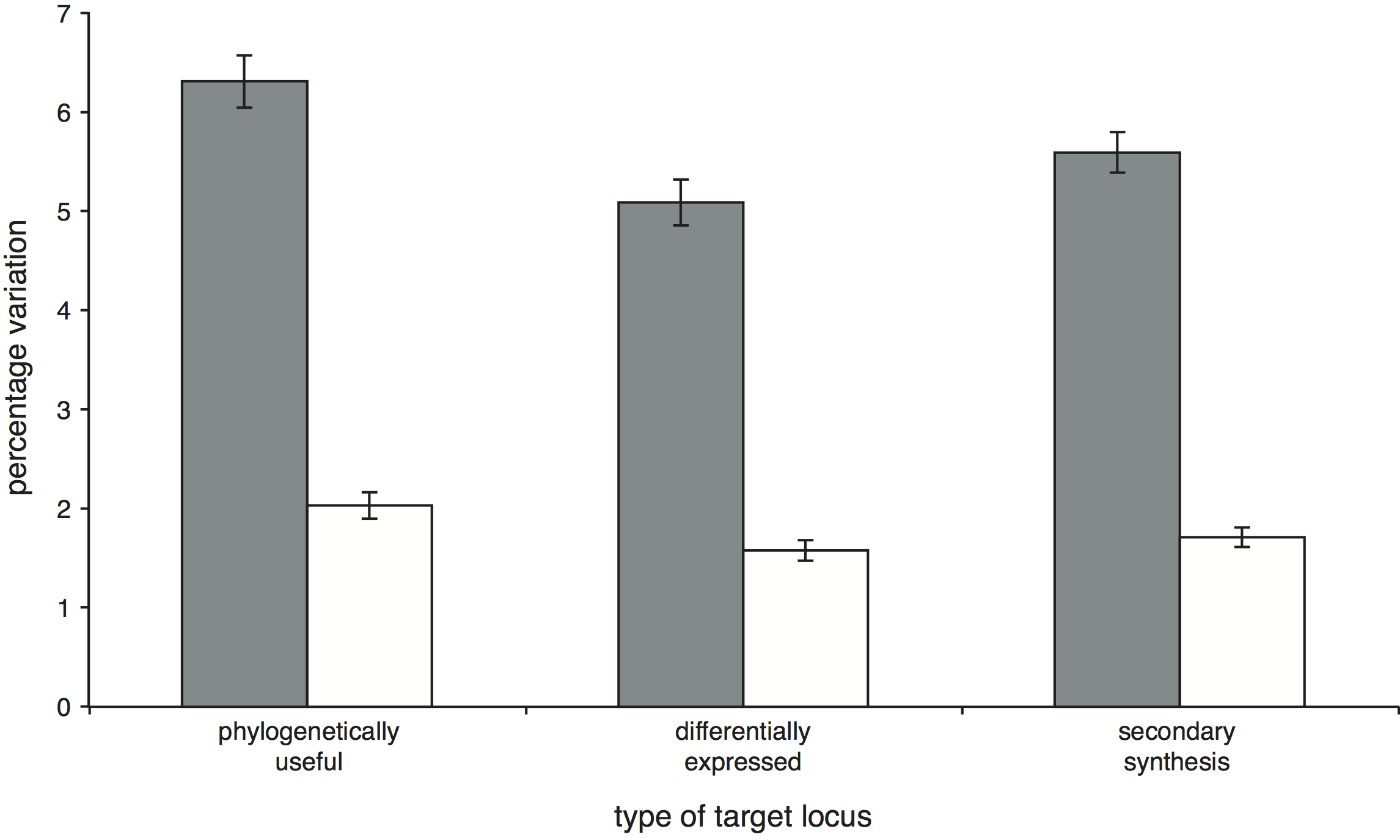

Supplement: Supplementary Figure 11 — Mean percentage (±SE) of variable (gray bars) and parsimony informative sites (white bars) across the three types of target loci. [file Image11.JPEG]

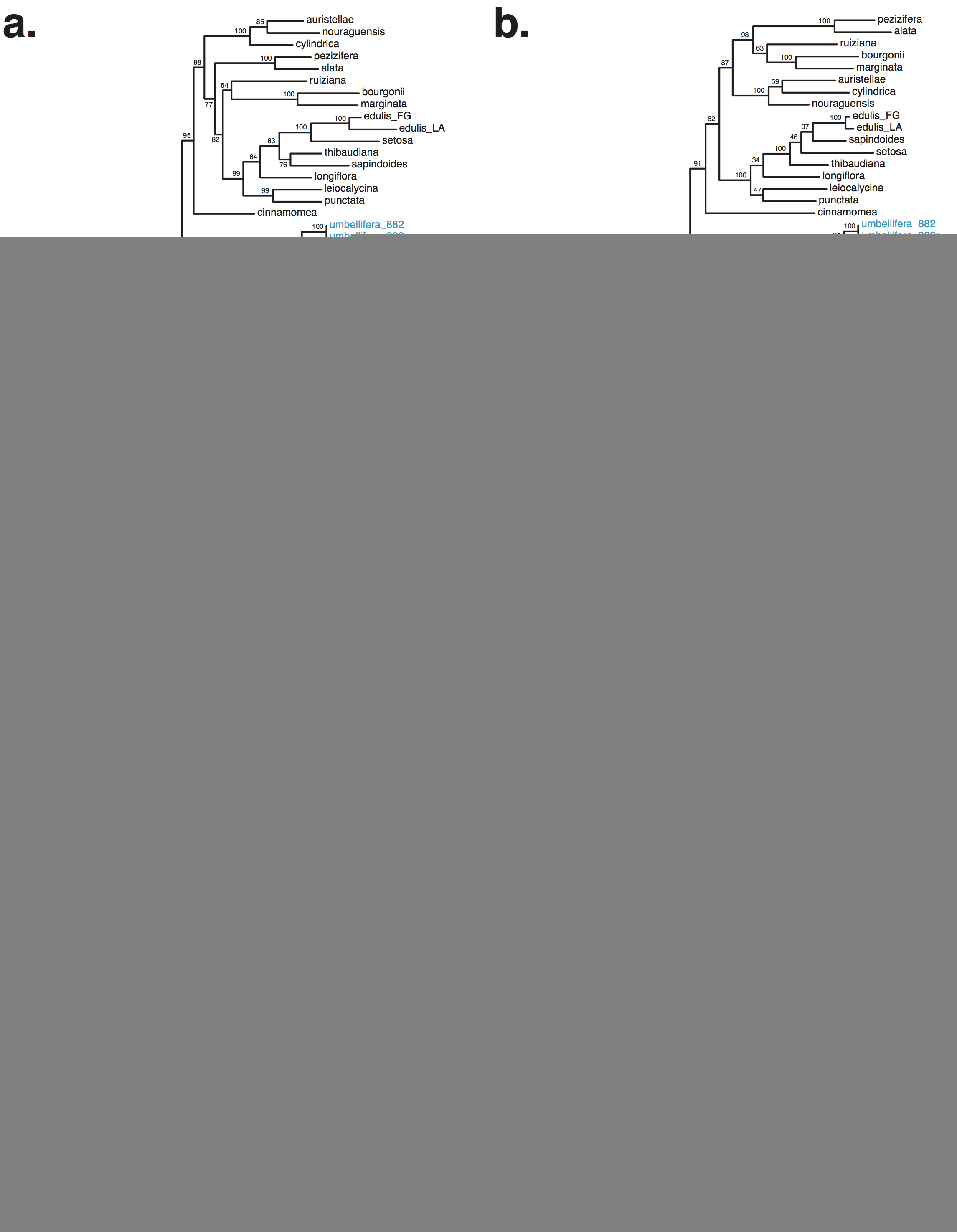

Supplement: Supplementary Figure 12 — Maximum likelihood phylogenies derived using the three different types of target loci. (A) Phylogenetically informative loci (30 loci, 45,648 bp); (B) differentially expressed loci (72 loci, 119,011 bp); (C) secondary synthesis pathway loci (66 loci, 111,455 bp); and (D) non-secondary synthesis loci (66 loci, 133,754 bp) consisting of 30 phylogenetically informative loci and 36 differentially expressed loci. Numbers next to nodes indicate bootstrap support. [file Image12.JPEG]
